# Integrative multi-omics analysis reveals CXCL10-driven inflammation and TREM2 + macrophage-plasma cell survival niche as hallmarks of late-stage rheumatoid arthritis

**DOI:** 10.1186/s13075-026-03764-3

**Published:** 2026-02-12

**Authors:** Jianbin Li, Mengxia Liu, Yilin Peng, Rui Wu

**Affiliations:** Department of Rheumatology and Immunology, The First Affiliated Hospital, Jiangxi Medical College, Nanchang University, No. 17 Yongwaizheng Street, Donghu District, Nanchang City, 330006 Jiangxi Province China

**Keywords:** Rheumatoid arthritis, CXCL10, Machine learning, Single-cell RNA sequencing, TREM2, Macrophages, Plasma cells, Biomarkers

## Abstract

**Background:**

Rheumatoid arthritis (RA) is characterized by persistent synovial inflammation, yet the molecular mechanisms distinguishing early from late-stage disease remain incompletely elucidated. Identifying stage-specific biomarkers and pathogenic cellular interactions is crucial for precision medicine.

**Objective:**

To comprehensively characterize the transcriptomic landscape and cellular composition of early versus late RA synovium, identify diagnostic biomarkers, and elucidate key pathogenic cell–cell interactions driving disease chronicity.

**Methods:**

Synovial tissues from 51 RA patients (13 early, 38 late-stage) were analyzed using histopathology, immunohistochemistry, bulk RNA sequencing (*n* = 19),and single-cell RNA sequencing (scRNA-seq, *n* = 6; 3 Early RA vs. 3 Late-stage RA).Machine learning algorithms (LASSO, SVM-RFE, random forest) were employed to identify diagnostic biomarkers. An artificial neural network (ANN) model was constructed and validated. Cell–cell communication analysis was performed using CellChat.

**Results:**

Histopathological analysis revealed significantly increased infiltration of macrophages (CD68 +) and plasma cells (CD138 +) in late-stage RA (*P* < 0.05). RNA sequencing identified 87 differentially expressed genes, with interferon-stimulated genes significantly upregulated. Integrated machine learning identified a minimal three-gene signature (CXCL10, ISG15, IFIH1) as a promising candidate model for RA staging. The three-gene ANN model showed excellent diagnostic performance (AUC = 0.922). Notably, CXCL10 emerged as the most critical component, demonstrating potentially high classification accuracy in this cohort (AUC = 0.767) and standing as the sole independent predictor in multivariable analysis (OR = 7.271, *P* = 0.022). CXCL10 high expression was strongly associated with M1 macrophage infiltration (*r* = 0.446, *P* = 0.005) and enriched in chemokine and JAK-STAT pathways. scRNA-seq revealed macrophages as the primary source of CXCL10, with upstream stimulation from CD8 + T cells via the IFN-γ-CXCL10-CXCR3 axis. Critically, we identified an expanded TREM2 + macrophage subset in late RA, which highly expressed APRIL (TNFSF13) and expanded in parallel with plasma cells expressing APRIL receptors (BCMA + /TACI +). This TREM2 + macrophage-plasma cell niche may represent a potential pathogenic circuit that could contribute to autoimmune chronicity.

**Conclusions:**

Late-stage RA appears to be characterized by a CXCL10-driven inflammatory signature and an expanded TREM2 + macrophage-plasma cell survival niche. CXCL10 represents a promising candidate biomarker for disease staging that may have mechanistic links to pathogenesis. The IFN-γ-CXCL10-CXCR3 axis and the APRIL-BCMA/TACI pathway may constitute potential therapeutic targets for refractory RA.

**Supplementary Information:**

The online version contains supplementary material available at 10.1186/s13075-026-03764-3.

## Introduction

Rheumatoid arthritis (RA) is a chronic systemic autoimmune disease affecting approximately 0.5–1% of the global population, characterized by persistent synovial inflammation leading to progressive joint destruction and disability [1, 2]. Despite significant therapeutic advances with biological and targeted synthetic disease-modifying antirheumatic drugs (b/tsDMARDs), approximately 30–40% of patients develop refractory disease, manifesting as persistent synovitis, progressive structural damage, and diminished quality of life [3, 4]. Understanding the molecular and cellular mechanisms underlying disease progression from early inflammatory arthritis to established, treatment-resistant RA remains a critical unmet need.

The synovial microenvironment in RA undergoes profound cellular and molecular remodeling during disease evolution. Early RA is typically characterized by acute inflammatory infiltration dominated by activated T cells and pro-inflammatory cytokines, whereas late-stage disease exhibits distinct features including persistent immune cell accumulation, fibroblast activation, and formation of organized lymphoid-like structures [5–7]. However, the precise transcriptomic signatures distinguishing these stages, the key cellular players driving chronicity, and their molecular interactions remain incompletely characterized.

Recent histopathological and molecular studies have highlighted the pivotal roles of macrophages and plasma cells in RA pathogenesis. Synovial macrophage numbers correlate strongly with disease activity and radiographic progression, with different macrophage subsets displaying pro-inflammatory (M1-like) or tissue-remodeling (M2-like) phenotypes [8, 9]. Plasma cells, as terminally differentiated effector cells of the B cell lineage, produce pathogenic autoantibodies including rheumatoid factor (RF) and anti-citrullinated protein antibodies (ACPA), and their accumulation in synovial tissue is associated with poor therapeutic response [10, 11]. Notably, recent studies have identified specialized macrophage subsets expressing triggering receptor expressed on myeloid cells 2 (TREM2) that may support plasma cell survival through secretion of the B cell survival factor APRIL (a proliferation-inducing ligand, encoded by TNFSF13) [12, 13]. However, whether this macrophage-plasma cell axis is specifically expanded in late-stage RA and how it relates to the broader inflammatory landscape remains unclear.

The advent of high-throughput transcriptomics, including bulk RNA sequencing (RNA-seq) and single-cell RNA sequencing (scRNA-seq), has revolutionized our understanding of complex inflammatory diseases. These technologies enable unbiased, genome-wide characterization of gene expression patterns and cellular heterogeneity at unprecedented resolution [9, 14]. When integrated with advanced computational approaches such as machine learning and cell–cell communication inference, multi-omics analyses can identify novel biomarkers and decode pathogenic cellular circuits [15, 16]. Such approaches are particularly valuable for complex diseases like RA, where traditional histology and immunohistochemistry provide limited mechanistic insights.

Despite these advances, several key questions remain unanswered: (1) What are the defining transcriptomic signatures distinguishing early and late-stage RA at both bulk tissue and single-cell levels? (2) Can robust molecular biomarkers be identified to accurately classify disease stage and predict treatment outcomes? (3) What are the key cellular players and molecular pathways driving the transition from acute inflammation to chronic, treatment-resistant synovitis? (4) How do different immune cell populations interact to maintain pathogenic inflammation in established disease?

To address these questions, we conducted an integrated, multi-modal analysis of synovial tissues from early and late-stage RA patients. We first performed comprehensive histopathological assessment and immunohistochemical characterization of immune cell infiltration. We then applied bulk RNA-seq to identify differentially expressed genes and dysregulated pathways, followed by machine learning-based feature selection to identify a minimal gene signature with maximal diagnostic power. To validate and extend these findings at single-cell resolution, we performed scRNA-seq to define cellular heterogeneity, characterize cell type-specific gene expression, and infer cell–cell communication networks. Finally, we explored potential therapeutic implications by conducting in silico drug screening and molecular docking analyses.

Our integrative approach reveals that late-stage RA is characterized by a CXCL10-driven dominant inflammatory signature and the emergence of a specialized TREM2 + macrophage-plasma cell survival niche. These findings provide new mechanistic insights into RA chronicity and identify potential therapeutic targets for treatment-resistant disease.

## Methods

### Study design and patient cohort

This study was designed as a prospective cross-sectional analysis aimed at comprehensively characterizing the molecular and cellular features of early versus late-stage RA synovium. Between January 2020 and December 2023, we recruited 51 RA patients from the Department of Rheumatology and Immunology at our institution. All patients fulfilled the 2010 American College of Rheumatology (ACR)/European League Against Rheumatism (EULAR) RA classification criteria [17]. The study was approved by the Institutional Ethics Committee, and all participants provided written informed consent before enrollment.Patients were classified into two groups based on disease duration and clinical characteristics:Early RA (*n* = 13): Disease duration ≤ 2 years from symptom onset, treatment-naïve or receiving only conventional synthetic DMARDs (csDMARDs), with no radiographic evidence of joint erosions on baseline X-rays.Late-stage RA (*n* = 38): Disease duration > 2 years, radiographic evidence of joint damage (Larsen score ≥ 2), and/or inadequate response to at least one csDMARD or biological DMARD, defined as persistent moderate-to-high disease activity (DAS28-CRP > 3.2) despite adequate treatment duration.

Demographic and clinical data collected at baseline included age, sex, disease duration, Disease Activity Score-28 using C-reactive protein (DAS28-CRP), swollen and tender joint counts (SJC28, TJC28), patient visual analog scale global assessment (VAS), erythrocyte sedimentation rate (ESR), C-reactive protein (CRP), rheumatoid factor (RF), and anti-citrullinated protein antibody (ACPA) status. Radiographic assessment was performed using standard anteroposterior X-rays of hands and feet, scored according to the Larsen grading system.From this cohort, a subset of 19 samples (10 Early, 9 Late) was selected for bulk RNA-seq, and 6 representative samples (3 Early, 3 Late) were processed for scRNA-seq analysis.

### Synovial tissue sampling

Synovial tissue biopsies were obtained using ultrasound-guided needle biopsy under local anesthesia from the most clinically active joint (defined by swelling, tenderness, and/or effusion). For each patient, 6–10 tissue cores were collected and immediately allocated as follows: 2–3 cores were fixed in 4% paraformaldehyde for histopathology and immunohistochemistry, 2–3 cores were snap-frozen in liquid nitrogen for RNA extraction, and 2–4 cores (if available) were placed in cold RPMI-1640 medium for immediate processing for single-cell RNA sequencing. Exclusion criteria included intra-articular corticosteroid injection within 4 weeks prior to biopsy, active infection, or other inflammatory arthropathies.

### Histopathology and immunohistochemistry

Formalin-fixed synovial tissues were paraffin-embedded and sectioned at 4 μm thickness. Sections were stained with hematoxylin and eosin (H&E) for morphological assessment. Synovial histopathological features including synovial lining hyperplasia, inflammatory cell infiltration, stromal activation, and neovascularization were semi-quantitatively scored (grade 0–3: 0 = absent, 1 = mild, 2 = moderate, 3 = marked) by two independent pathologists blinded to clinical data, with discrepancies resolved by consensus.Immunohistochemical staining was performed using the avidin–biotin-peroxidase complex method. Briefly, sections were deparaffinized, rehydrated, and subjected to heat-induced antigen retrieval in citrate buffer (pH 6.0). Endogenous peroxidase activity was blocked with 3% hydrogen peroxide. Sections were incubated overnight at 4 °C with primary antibodies including anti-CD3 (T cells), CD20 (B cells), CD68 (pan-macrophage marker), and CD138 (plasma cells). After washing, sections were incubated with biotinylated secondary antibody followed by streptavidin–horseradish peroxidase complex. Visualization was performed using 3,3'-diaminobenzidine (DAB) chromogen, and sections were counterstained with hematoxylin. Immune cell infiltration was assessed by scoring positive cell density in representative high-power fields (× 400 magnification) and categorized as enrichment groups (0 = absent/minimal, 1 = moderate enrichment defined as > 5% but < 20% infiltrating cells, 2 = high enrichment defined as ≥ 20% infiltrating cells).

### Bulk RNA sequencing and bioinformatics analysis

#### RNA extraction and library preparation

Total RNA was extracted from snap-frozen synovial tissues using TRIzol reagent (Invitrogen, USA) according to the manufacturer's protocol. RNA quality and concentration were assessed using NanoDrop spectrophotometry and an Agilent 2100 Bioanalyzer. Only samples with RNA integrity number (RIN) ≥ 7.0 and 260/280 ratio between 1.8–2.0 were used for library preparation. RNA-seq libraries were constructed using the VAHTS Universal V6 RNA-seq Library Prep Kit (Vazyme, China) according to manufacturer's instructions. Briefly, mRNA was enriched using oligo(dT) magnetic beads, fragmented, and reverse transcribed into cDNA. Following end repair, adapter ligation, and PCR amplification, libraries were purified and quantified. Sequencing was performed on an Illumina NovaSeq 6000 platform with 150 bp paired-end reads, targeting approximately 40 million reads per sample.

#### Read processing and differential expression analysis

Raw sequencing reads were processed using fastp (v0.23.2) to remove adapter sequences and low-quality bases (quality score < 20). Clean reads were aligned to the human reference genome (GRCh38/hg38) using HISAT2 (v2.2.1), and gene-level read counts were quantified using featureCounts in the Subread package (v2.0.3). Reads mapping to ribosomal RNA, mitochondrial genes, and pseudogenes were excluded from downstream analysis.

Differential gene expression analysis between early and late RA groups was performed using the limma package in R (v4.2.0). First, expression values for duplicate probes were averaged (avereps function). Subsequently, design and contrast matrices were constructed (model.matrix and makeContrasts), and linear models were fitted and Bayesian statistics calculated using lmFit, contrasts.fit, and eBayes. Differentially expressed genes (DEGs) were defined using the following criteria: |log2(fold change)|> 0.58 (corresponding to a > 1.5-fold change) and nominal *P* value < 0.01. Volcano plots and heatmaps were generated using the ggplot2 and pheatmap packages, respectively.

#### Functional enrichment analysis

Gene Ontology (GO) and Kyoto Encyclopedia of Genes and Genomes (KEGG) pathway enrichment analyses were performed using the clusterProfiler package (v4.4.4) in R. Enrichment analysis was performed using the hypergeometric test. To comprehensively capture potential biological insights and avoid excluding biologically relevant pathways, significance was defined as a nominal P value < 0.05. For visualization, we displayed the top enriched terms ranked by nominal P values and reported the corresponding false discovery rates (FDR). Gene Set Enrichment Analysis (GSEA) was performed using GSEA software (v4.3.2) from the Broad Institute with gene sets from the Molecular Signatures Database (MSigDB) v7.5. Normalized enrichment scores (NES) and false discovery rates (FDR q-values) were calculated using 1,000 permutations.

#### Protein–protein interaction network and hub gene identification

A protein–protein interaction (PPI) network was constructed for DEGs using the STRING database (v11.5) with a minimum required interaction score of 0.4 (medium confidence). The network was visualized and analyzed using Cytoscape (v3.9.1). To identify hub genes with high topological importance, we applied four complementary algorithms implemented in the cytoHubba plugin: Degree centrality (Degree), Edge Percolated Component (EPC), Maximal Clique Centrality (MCC), and Maximum Neighborhood Component (MNC). The top 20 genes identified by each method were extracted, and genes ranked in the top 20 by all four algorithms were defined as core hub genes.

### Machine learning-based feature selection

To identify a minimal gene signature with maximal diagnostic power for RA staging, we applied three independent machine learning algorithms to the core hub genes:

#### Least Absolute Shrinkage and Selection Operator (LASSO) regression

LASSO logistic regression was implemented using the glmnet package (v4.1–6) in R. The optimal regularization parameter (λ) was determined through tenfold cross-validation, selecting the λ value that minimized binomial deviance. Genes with non-zero coefficients at the optimal λ were retained as LASSO-selected features.

#### Support Vector Machine-Recursive Feature Elimination (SVM-RFE)

SVM-RFE was performed using the e1071 (v1.7–12) and caret (v6.0–93) packages in R. The algorithm iteratively built SVM models, ranked features based on their contribution to classification accuracy, and recursively eliminated the least important features. Ten-fold cross-validation was employed at each iteration to evaluate model performance. The feature subset yielding the lowest cross-validation error and highest accuracy was selected.

#### Random Forest (RF)

RF analysis was conducted using the randomForest package (v4.7–1.1) in R. The model was trained with 500 decision trees (ntree = 500) and default mtry parameter (square root of the number of features). Feature importance was quantified using mean decrease in Gini index, which measures total decrease in node impurity from splitting on each feature across all trees. Features were ranked by importance, and the highest-scoring features were selected.

The final core signature genes were defined as those identified by all three machine learning methods, representing a robust, algorithm-independent gene signature.

### Diagnostic model construction and validation

#### Artificial Neural Network (ANN) Model

An ANN classification model was constructed using the neuralnet package (v1.44.2) in R based on expression levels of the final core signature genes. The network architecture consisted of three layers: an input layer with nodes equal to the number of signature genes, a single hidden layer containing three nodes, and an output layer with two nodes representing the two disease stages. The model was trained using the resilient backpropagation algorithm with weight backtracking. Training was performed for a maximum of 10,000 iterations with a learning rate threshold of 0.01.

#### Nomogram construction

A clinical nomogram was developed through logistic regression using the rms package (v6.3–0) in R. The nomogram integrated expression levels of the core signature genes to generate individualized risk predictions for late-stage RA. Model calibration was assessed using calibration plots comparing predicted probabilities with observed outcomes, with 1,000 bootstrap resamples for overfitting correction. Clinical utility was evaluated using decision curve analysis (DCA), calculating net benefit across a range of threshold probabilities, and generating a clinical impact curve (CIC) to visualize the number of high-risk individuals and true positives across different risk thresholds.

#### Receiver Operating Characteristic (ROC) analysis

ROC curves were generated for the ANN model and individual signature genes using the pROC package (v1.18.0) in R. Area under the curve (AUC) values with 95% confidence intervals were calculated using the DeLong method. Optimal cutoff values, sensitivity, and specificity were determined using the Youden index.

### Immune cell infiltration analysis

To characterize the immune cell composition of synovial tissue from bulk RNA-seq data, we applied the CIBERSORT algorithm (v1.04), which uses support vector regression to deconvolute the relative proportions of 22 immune cell types from mixed tissue gene expression profiles. CIBERSORT was run using the LM22 signature matrix with 1,000 permutations. Samples with CIBERSORT *P* < 0.05 were considered to have reliable deconvolution results and included in downstream analyses. Wilcoxon rank-sum test was used to assess differences in cell type proportions between early and late RA. Spearman correlation analysis was performed to evaluate associations between core signature gene expression levels and immune cell subset abundances.

### Single-Cell RNA Sequencing (scRNA-seq)

#### Single-cell suspension preparation

Fresh synovial tissues were minced into small pieces (< 1 mm3) and enzymatically digested in RPMI-1640 medium containing 1 mg/mL collagenase IV (Sigma-Aldrich) and 0.1 mg/mL DNase I (Roche) at 37 °C with gentle agitation for 60 min. The digested suspension was filtered through a 70 μm cell strainer (Corning) to remove tissue debris. Red blood cells were lysed using ACK lysis buffer (Gibco) for 5 min at room temperature. Cells were washed twice with phosphate-buffered saline (PBS) containing 0.04% bovine serum albumin (BSA), resuspended to approximately 1,000 cells/μL concentration, and viability assessed using trypan blue exclusion. Only samples with viability > 80% were processed for scRNA-seq.

#### Library preparation and sequencing

Single-cell libraries were prepared using the 10 × Genomics Chromium Single Cell 3' Gene Expression platform (v3.1 chemistry) according to the manufacturer's protocol. Briefly, single-cell suspensions were loaded onto the Chromium Controller to generate gel bead-in-emulsions (GEMs) where individual cells are encapsulated with barcoded gel beads and lysis reagents. Following reverse transcription and cDNA amplification, libraries were constructed and sequenced on an Illumina NovaSeq 6000 platform, targeting approximately 50,000 reads per cell.

#### Data preprocessing and quality control

Raw sequencing data were processed using Cell Ranger (v6.1.2, 10 × Genomics) for demultiplexing, alignment to the GRCh38 reference genome, and generation of gene-barcode matrices. Downstream analysis was performed using the Seurat package (v4.3.0) in R. Quality control filtering was applied to remove low-quality cells based on the following criteria: (1) cells with < 200 or > 6,000 detected genes (nFeature_RNA), (2) cells with < 500 unique molecular identifiers (nCount_RNA), (3) cells with > 20% mitochondrial gene content (percent.mt). Additionally, potential doublets were identified and removed using the DoubletFinder package (v2.0.3).

#### Dimensionality reduction, clustering, and cell type annotation

After quality control, gene expression data were normalized using log normalization (NormalizeData function), and the top 2,000 highly variable genes were identified using the FindVariableFeatures function. Data were scaled (ScaleData function), and batch effects were corrected using the Harmony algorithm (v0.1.1) when integrating samples from different patients. Principal component analysis (PCA) was performed on scaled data, and the first 30 principal components (PCs) were used for downstream analysis based on elbow plot inspection.Cells were clustered using the Louvain algorithm based on shared nearest neighbor (SNN) graphs (FindNeighbors and FindClusters functions) with a resolution parameter of 0.5. t-distributed stochastic neighbor embedding (t-SNE) was used for non-linear dimensionality reduction for visualization. Cell type annotation was performed using canonical marker genes: CD14, CD68, and FCGR3A for myeloid cells; CD3D, CD3E, and CD8A for T cells; CD79A, MS4A1 (CD20), and CD19 for B cells; MZB1, IGHG1, and SDC1 (CD138) for plasma cells; COL1A1, COL3A1, and DCN for fibroblasts; PECAM1 (CD31) and VWF for endothelial cells. Cluster annotations were manually inspected and refined based on differentially expressed marker genes identified using the FindAllMarkers function.

#### Macrophage subset analysis

Macrophage clusters were extracted from the integrated Seurat object based on established marker gene expression (e.g., CD14, CD68, LYZ). Secondary clustering analysis was then performed using the standard Seurat workflow with a higher resolution parameter (resolution = 0.8) to resolve finer-grained subsets. Differential expression analysis for subsets was conducted using the FindAllMarkers function (parameters: only.pos = TRUE, min.pct = 0.25, logfc.threshold = 0.5). TREM2 + macrophage subsets were identified based on TREM2 expression (log-normalized expression > 0). Expression of key genes including TREM2, SPP1, and TNFSF13 (APRIL) was visualized using violin plots and feature plots. Differential abundance testing between early and late RA samples was performed using the propeller function in the speckle package (v1.0.0), which accounts for inter-sample variability and compositional effects.

#### Trajectory analysis

Pseudotime trajectory analysis was performed using Monocle 3 (v1.0.0) to infer developmental relationships and differentiation paths. Cells were ordered along trajectories based on gene expression dynamics, with root states manually defined based on known progenitor markers. For macrophage differentiation analysis, classical CD14 + monocytes were designated as the trajectory root. For B cell to plasma cell differentiation, naïve B cells were used as the starting point. Genes dynamically expressed along trajectories were identified and visualized as heatmaps showing temporal expression patterns.

#### Cell–cell communication analysis

Cell–cell communication networks were inferred using CellChat (v1.1.3), which quantifies communication probability between cell types based on expression of ligands, receptors, and their cofactors from a curated signaling database. CellChat analysis was performed separately for early and late RA samples to identify stage-specific communication patterns. Ligand-receptor pairs were identified, and communication strength was calculated. Differential communication analysis was performed to identify interactions enriched in late-stage disease. Chord diagrams and circle plots were generated to visualize communication networks, with focus on the CXCL and APRIL signaling pathways.

### Statistical analysis

Continuous variables are presented as mean ± standard deviation or median with interquartile range as appropriate. Categorical variables are presented as frequencies and percentages. Group comparisons for continuous variables were performed using Student's t-test or Mann–Whitney U test depending on data distribution. Categorical variables were compared using Chi-square test or Fisher's exact test. Logistic regression was used to identify independent predictors, with odds ratios (OR) and 95% confidence intervals (CI) calculated. Correlation analyses were performed using Spearman's rank correlation coefficient. All statistical tests were two-sided, and *P* < 0.05 was considered statistically significant. Statistical analyses were performed using R (v4.2.0) and GraphPad Prism (v9.0).

## Results

### Baseline characteristics

Table 1 details the baseline clinical information and histopathological features of the 51 RA patients (13 early RA, 38 late-stage RA). No significant differences were observed between the two groups in terms of sex (*p* = 0.598), age (*p* = 0.695), or serological markers including ACPA (*p* = 0.542), ESR (*p* = 0.633), and RF (*p* = 0.509). However, as expected, the late RA group had significantly longer disease duration compared to the early RA group (*p* = 0.001). Notably, the early RA group exhibited higher inflammatory activity, with significantly elevated CRP levels compared to the late RA group (*p* = 0.031). At the histopathological level, features such as synovial hyperplasia, stromal activation, and neovascularization assessed by H&E staining showed no statistical differences between groups (*p* > 0.05), nor did the infiltration levels of T cells (CD3) and B cells (CD20). Key differences were manifested in macrophage and plasma cell infiltration: the proportion of patients with macrophage (CD68group, *p* = 0.019) and plasma cell (CD138group, *p* = 0.003) enrichment was significantly higher in the late RA group compared to the early RA group.

**Table 1:** Baseline patient characteristics

Characteristic	Total (*n* = 51)	Early (*n* = 13)	Late (*n* = 38)	*P* value
Gender, Female, *n* (%)	45 (88.2%)	12 (92.3%)	33 (86.8%)	0.598
Age (years)	53.00 ± 11.30	51.92 ± 6.14	53.37 ± 12.64	0.695
ACPA (U/mL)	150.95 ± 243.42	115.06 ± 229.60	163.56 ± 249.91	0.542
Disease duration (months)	88.00 ± 80.16	7.38 ± 4.07	115.58 ± 74.93	0.001
ESR (mm/h)	45.67 ± 30.05	49.15 ± 34.98	44.47 ± 28.59	0.633
CRP (mg/L)	36.63 ± 36.33	56.13 ± 37.84	30.47 ± 34.06	0.031
SJC28	5.08 ± 3.40	6.31 ± 3.92	4.66 ± 3.15	0.133
TJC28	5.55 ± 3.39	7.08 ± 3.68	5.03 ± 3.17	0.059
VAS	65.59 ± 11.16	69.23 ± 9.75	64.34 ± 11.45	0.175
DAS28	4.78 ± 1.01	5.22 ± 1.13	4.62 ± 0.93	0.067
RF (IU/mL)	210.67 ± 375.22	150.62 ± 267.24	231.22 ± 406.69	0.509
X-ray grade, *n* (%)				0.062
0	17 (33.3%)	8 (61.5%)	9 (23.7%)	
1	2 (3.9%)	0 (0.0%)	2 (5.3%)	
2	23 (45.1%)	4 (30.8%)	19 (50.0%)	
3	5 (9.8%)	0 (0.0%)	5 (13.2%)	
Synovial hyperplasia, *n* (%)				0.678
0	18 (35.3%)	4 (30.8%)	14 (36.8%)	
1	16 (31.4%)	4 (30.8%)	12 (31.6%)	
2	7 (13.7%)	3 (23.1%)	4 (10.5%)	
3	1 (2.0%)	0 (0.0%)	1 (2.6%)	
Inflammatory infiltration, *n* (%)				0.441
1	13 (25.5%)	4 (30.8%)	9 (23.7%)	
2	24 (47.1%)	5 (38.5%)	19 (50.0%)	
3	4 (7.8%)	2 (15.4%)	2 (5.3%)	
Stromal activity, *n* (%)				0.670
0	2 (3.9%)	0 (0.0%)	2 (5.3%)	
1	28 (54.9%)	8 (61.5%)	20 (52.6%)	
2	12 (23.5%)	3 (23.1%)	9 (23.7%)	
Neovascularization, *n* (%)				0.629
0	2 (3.9%)	1 (7.7%)	1 (2.6%)	
1	14 (27.5%)	3 (23.1%)	11 (28.9%)	
2	11 (21.6%)	2 (15.4%)	9 (23.7%)	
3	14 (27.5%)	5 (38.5%)	9 (23.7%)	
CD3 enrichment, *n* (%)	16 (31.4%)	3 (23.1%)	13 (34.2%)	0.551
CD20 enrichment, *n* (%)	14 (27.5%)	2 (15.4%)	12 (31.6%)	0.259
CD68 enrichment, *n* (%)	22 (43.1%)	2 (15.4%)	20 (52.6%)	0.019
CD138 enrichment, *n* (%)	33 (64.7%)	4 (30.8%)	29 (76.3%)	0.003

Data are presented as mean ± SD for continuous variables and *n* (%) for categorical variables. *P* values were calculated using independent t-tests for continuous variables and Chi-square or Fisher's exact tests for categorical variables

### Logistic regression analysis: predictive value of CD68 and CD138

Table 2 presents the results of logistic regression analysis for predicting late-stage RA. After adjusting for age and gender, multivariable analysis revealed that CRP levels (OR = 0.981, *p* = 0.037), macrophage enrichment (CD68 group, OR = 6.025, *p* = 0.032), and plasma cell enrichment (CD138 group, OR = 8.143, *p* = 0.004) were all significant independent predictors of late-stage RA. Among these, plasma cell enrichment (CD138 group) demonstrated the strongest predictive value with an OR of 8.143.

**Table 2:** Logistic regression analysis

Characteristic	Univariable		Multivariable	
	OR (95% CI)	*P*	OR (95% CI)	*P*
Age (years)	1.012 (0.957–1.070)	0.688	-	-
Gender (Male)	0.550 (0.058–5.199)	0.602	-	-
CRP	0.982 (0.965–0.999)	0.041	0.981 (0.963–0.999)	0.037
CD68 enrichment	6.111 (1.919–31.368)	0.03	6.025 (1.169–31.036)	0.032
CD138 enrichment	7.250 (1.796–29.258)	0.005	8.143 (1.941–34.157)	0.004

### Transcriptomic characteristics of late-stage RA synovial tissue

**Fig. 1:**
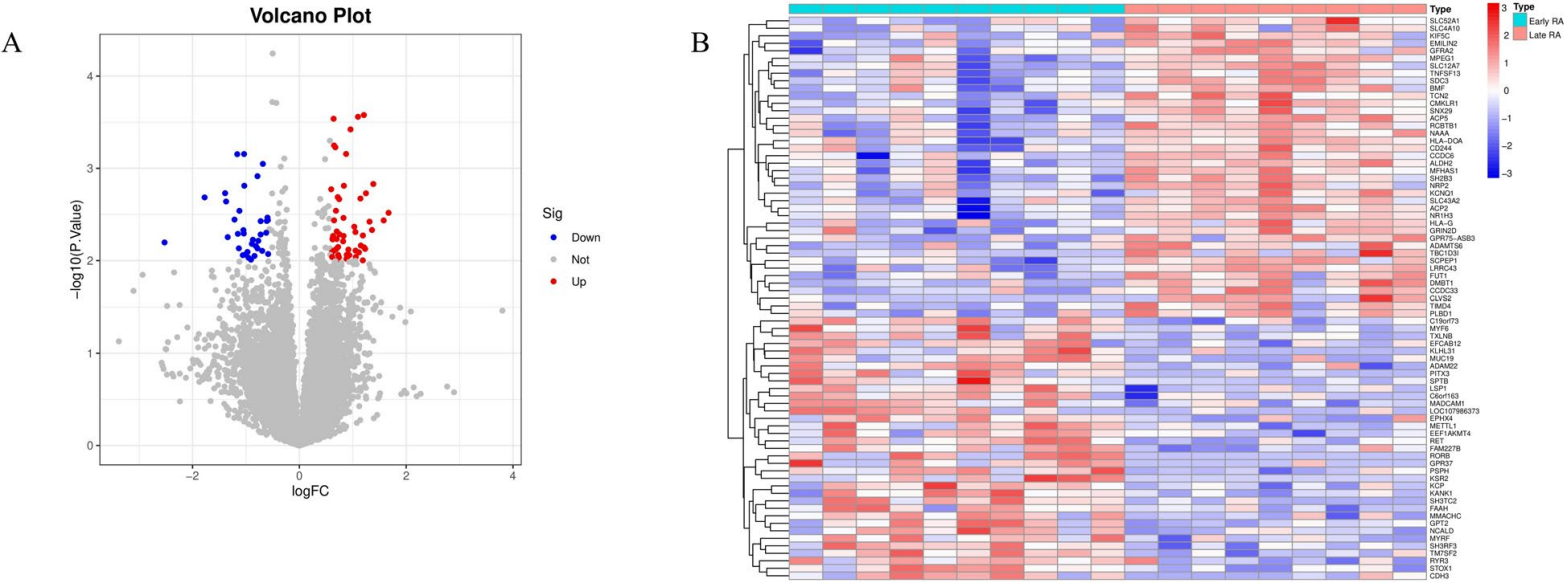
Differential gene expression analysis in late-stage rheumatoid arthritis synovial tissue. **A** Volcano plot of differentially expressed genes. Comparison of gene expression between late-stage RA (Late RA) and early RA (Early RA) synovial tissues. Red dots represent significantly upregulated genes, blue dots represent significantly downregulated genes, and gray dots represent non-significantly differentially expressed genes. **B** Heatmap of differentially expressed genes. Displays the Z-score normalized expression profiles of DEGs in early and late-stage RA samples. Rows represent genes, columns represent samples. Red indicates high expression, blue indicates low expression

To systematically explore the molecular differences between early RA and late-stage RA synovial tissues, we first performed whole-transcriptome RNA sequencing (bulk RNA-seq) analysis on 19 synovial samples (10 Early RA vs. 9 Late-stage RA).Differential expression analysis (DEG) results showed that compared to early RA, a total of 87 significantly differentially expressed genes were identified in late-stage RA synovial tissue, with 52 genes significantly upregulated and 35 genes significantly downregulated. The volcano plot (Fig. 1A) intuitively displays the distribution pattern of these DEGs. Heatmap analysis (Fig. 1B) further confirmed that the expression profiles of DEGs could clearly cluster early and late-stage RA samples into two distinct groups, showing markedly different molecular signatures, with late-stage RA samples exhibiting stronger immune activation-related gene expression characteristics.

### Functional enrichment analysis of differentially expressed genes

**Fig. 2:**
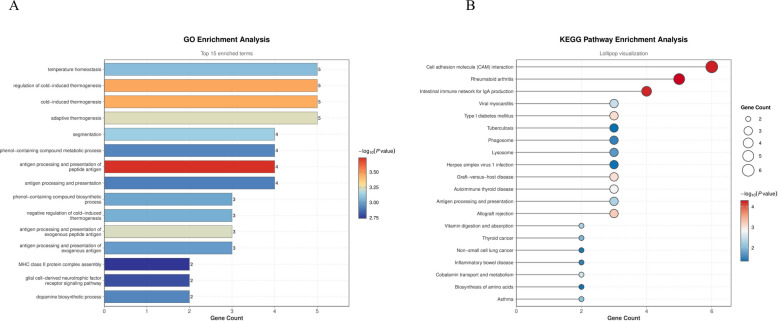
Functional enrichment analysis of differentially expressed genes (DEGs). **A** Bar chart of GO biological process (BP) enrichment analysis for differentially expressed genes. The figure displays the top 15 significantly enriched terms ranked by *P*-value, with the x-axis representing gene count and color representing significance level (-log10(P-value)). **B** Lollipop chart of KEGG pathway enrichment analysis for differentially expressed genes. The figure displays significantly enriched classical biological pathways and related disease pathways, with the x-axis representing the number of genes enriched in each pathway, bubbles

To elucidate the biological functions of differentially expressed genes, we performed GO and KEGG enrichment analyses. GO analysis results (Fig. 2A) showed that these genes were mainly enriched in biological processes related to temperature homeostasis, thermogenesis (including adaptive thermogenesis and cold-induced thermogenesis), and antigen processing and presentation of exogenous peptide antigens. Additionally, enrichment terms also involved MHC class II protein complex assembly and dopamine biosynthetic processes. KEGG pathway analysis (Fig. 2B) further confirmed significant enrichment of these genes in antigen processing and presentation, phagosome, and lysosome pathways, while also involving multiple immune-related diseases such as rheumatoid arthritis, type I diabetes mellitus, and allograft rejection.

### Protein–protein interaction network analysis and identification of core hub genes

**Fig. 3:**
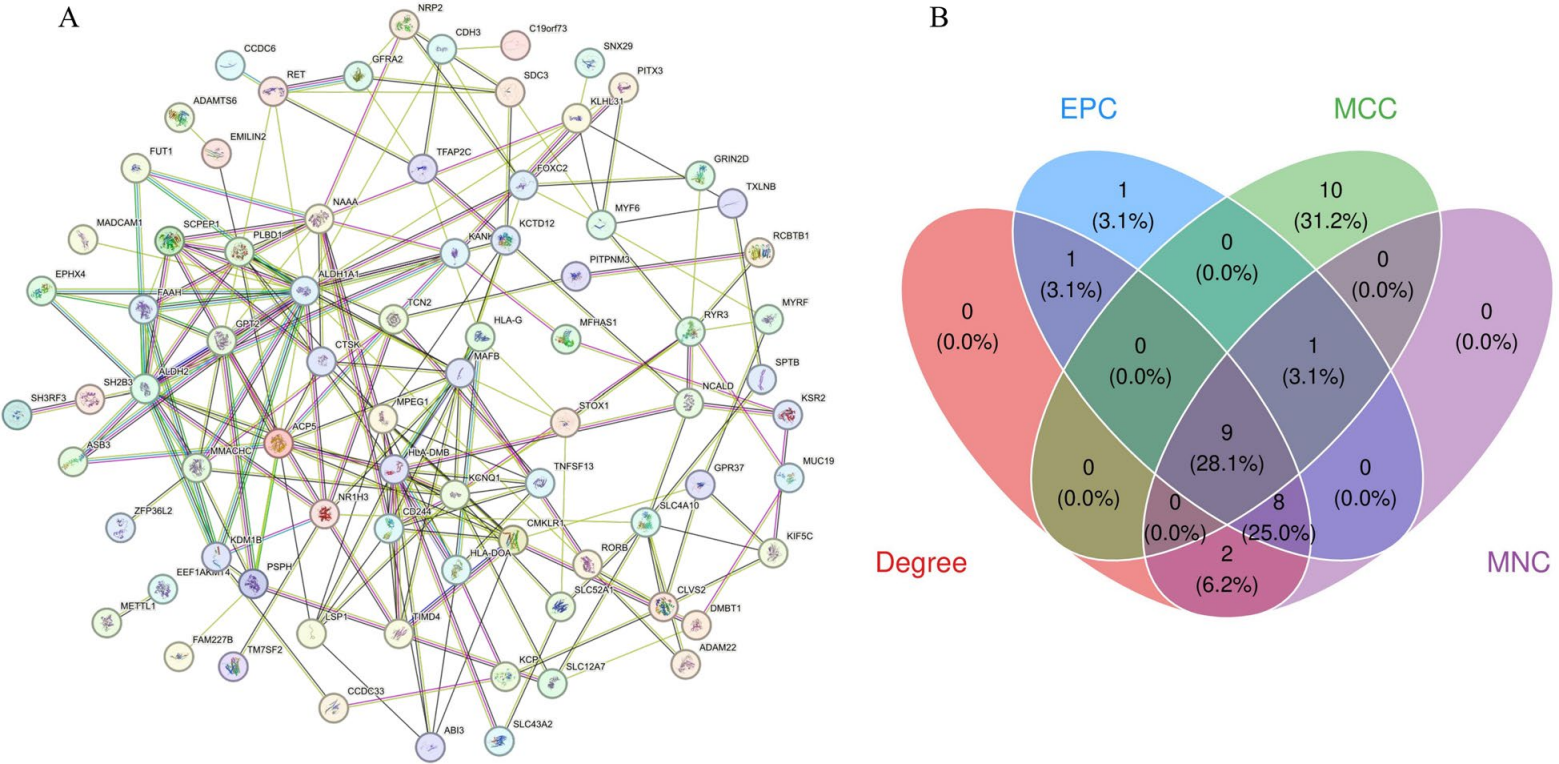
Identification of core hub genes based on protein-protein interaction network. **A** Protein-protein interaction (PPI) network diagram constructed from 87 differentially expressed genes (DEGs). Nodes represent proteins, edges represent protein-protein interaction relationships. **B** Venn diagram showing the intersection of the top 20 genes selected by four topological analysis algorithms (Degree, EPC, MCC, MNC). The central number 9 represents the core hub genes commonly identified by all four algorithms

To identify hub genes with core regulatory functions from the 87 differentially expressed genes (DEGs), we first constructed a protein–protein interaction (PPI) network. Figure 3A visualizes this network using the STRING database and Cytoscape software, where nodes represent proteins encoded by DEGs and edges represent their interaction relationships.To select the topologically most important nodes from the network, we further employed four different algorithms (Degree, EPC, MCC, and MNC) for analysis. As shown in Fig. 3B, the Venn diagram compared the top 20 genes selected by the four algorithms, showing that 9 genes were shared by all four methods. These 9 genes possess the highest topological centrality in the network and are considered the most robust core hub genes in the RA progression network, which will be used for subsequent machine learning modeling.

### Machine learning-based feature gene selection for RA staging

**Fig. 4:**
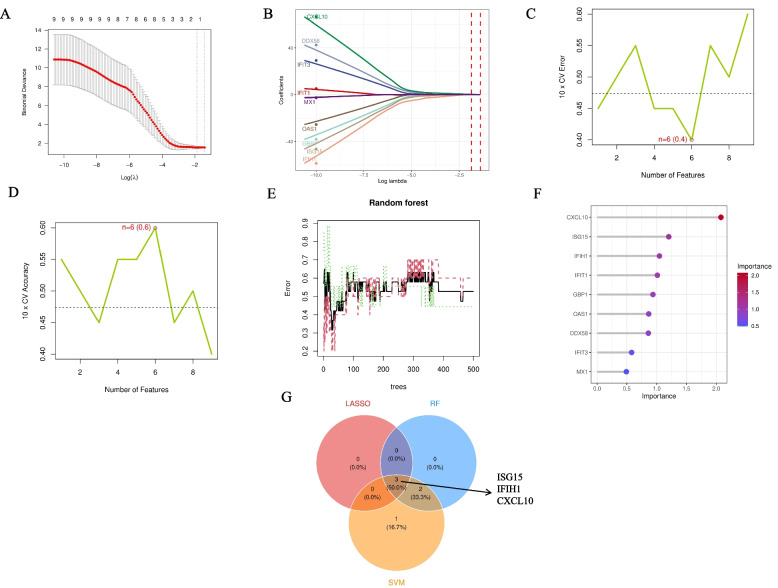
Integration of multiple machine learning algorithms for screening core feature genes for RA staging. **A** 10-fold cross-validation curve for LASSO regression, Y-axis is binomial deviance, X-axis is Log(λ). **B** LASSO regression coefficient path plot, showing the compression process of gene coefficients with changing penalty parameter log(λ). **C** SVM-RFE 10-fold cross-validation error curve, showing minimum error at 6 features. **D** SVM-RFE 10-fold cross-validation accuracy curve, showing highest accuracy at 6 features. **E** Random forest model error convergence plot. **F** Random forest feature importance ranking plot, CXCL10 scored highest. **G** Venn diagram of LASSO, random forest (RF), and support vector machine (SVM) screening results. The intersection shows 3 common feature genes: ISG15, IFIH1, and CXCL10

To further identify the most diagnostically valuable feature biomarkers from the 9 core hub genes selected in the previous step, we employed three independent machine learning algorithms for feature selection.First, LASSO regression analysis determined the optimal λ value through tenfold cross-validation (Fig. 4A) and selected 7 genes with non-zero coefficients, including CXCL10, DDX58, IFIH1, IFIT1, MX1, OAS1, and ISG15 (Fig. 4B).Second, we applied the support vector machine recursive feature elimination (SVM-RFE) algorithm. The tenfold cross-validation curve showed that when the number of features was 6, the model’s cross-validation error was lowest (CV Error = 0.4, Fig. 4C), with accuracy reaching its peak (Accuracy = 0.6, Fig. 4D).Finally, the random forest (RF) analysis error curve showed model stabilization (Fig. 4E). Its feature importance ranking plot (Fig. 4F) provided a key finding: CXCL10 scored highest among all genes, followed by ISG15 and IFIH1. This result suggests for the first time that CXCL10 may be the most important feature for distinguishing RA stages.The Venn diagram (Fig. 4G) integrated the results of the three algorithms, showing that ISG15, IFIH1, and CXCL10 were the only core feature genes commonly identified by all three machine learning methods. Therefore, these three genes were determined as the final biomarkers for our subsequent diagnostic modeling.

### Construction of neural network diagnostic model based on core feature genes

**Fig. 5:**
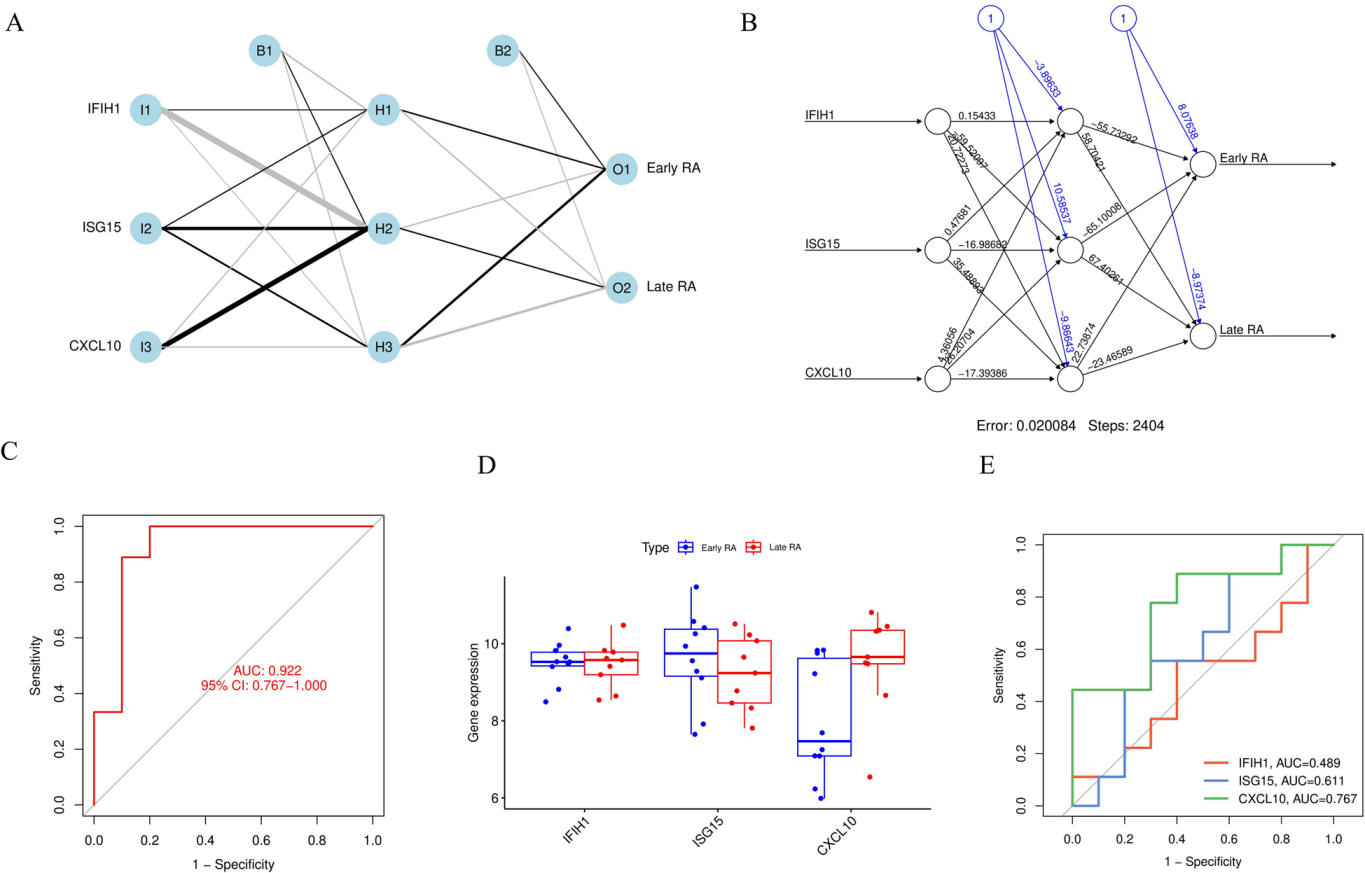
Construction and validation of artificial neural network diagnostic model based on three-gene signature. **A** Schematic diagram of ANN model architecture. **B** Network weight diagram after ANN model training, showing training error of 0.020084 and 2404 steps. **C** ROC curve based on three-gene combined ANN model, AUC of 0.922. **D** Box plots of IFIH1, ISG15, and CXCL10 expression levels in early RA (blue) and late-stage RA (red) samples. **E** Comparison of ROC curves for individual genes: IFIH1 (AUC=0.489), ISG15 (AUC=0.611), and CXCL10 (AUC=0.767)

Based on the three core feature genes (IFIH1, ISG15, CXCL10) selected by machine learning, we constructed an artificial neural network (ANN) diagnostic model to distinguish between early and late-stage RA.Fig. 5A and B display the ANN model architecture and trained weights. The model contains 3 input nodes, 3 hidden layer nodes, and 2 output nodes. The trained weight diagram (Fig. 5B) shows that ISG15 (I2) and CXCL10 (I3) both obtained extremely strong connection weights (−16.98 and −17.39, respectively), indicating that the model highly relies on the expression values of these two genes during decision-making.To evaluate the diagnostic performance of this three-gene model, we plotted its ROC curve. Results showed that the ANN model demonstrated excellent performance in distinguishing RA stages, with an area under the curve (AUC) of 0.922 (95% CI: 0.767–1.000) (Fig. 5C).We further validated the expression levels of these three genes in samples from different stages. Box plots (Fig. 5D) showed that IFIH1, ISG15, and CXCL10 expression levels were all significantly higher in late-stage RA patients compared to early RA.Finally, we compared the diagnostic ability of the combined model versus individual genes (Fig. 5E). Analysis found that CXCL10 was the best-performing single gene (AUC = 0.767), significantly superior to ISG15 (AUC = 0.611) and IFIH1 (AUC = 0.489). Nevertheless, the three-gene combined model (AUC = 0.922) significantly outperformed any single gene, demonstrating the superiority of multi-gene integrated models.

### Construction of clinical diagnostic nomogram model based on core genes

To translate the three-gene signature (IFIH1, ISG15, CXCL10) into a clinically applicable diagnostic tool, we constructed a logistic regression-based nomogram model.

**Fig. 6:**
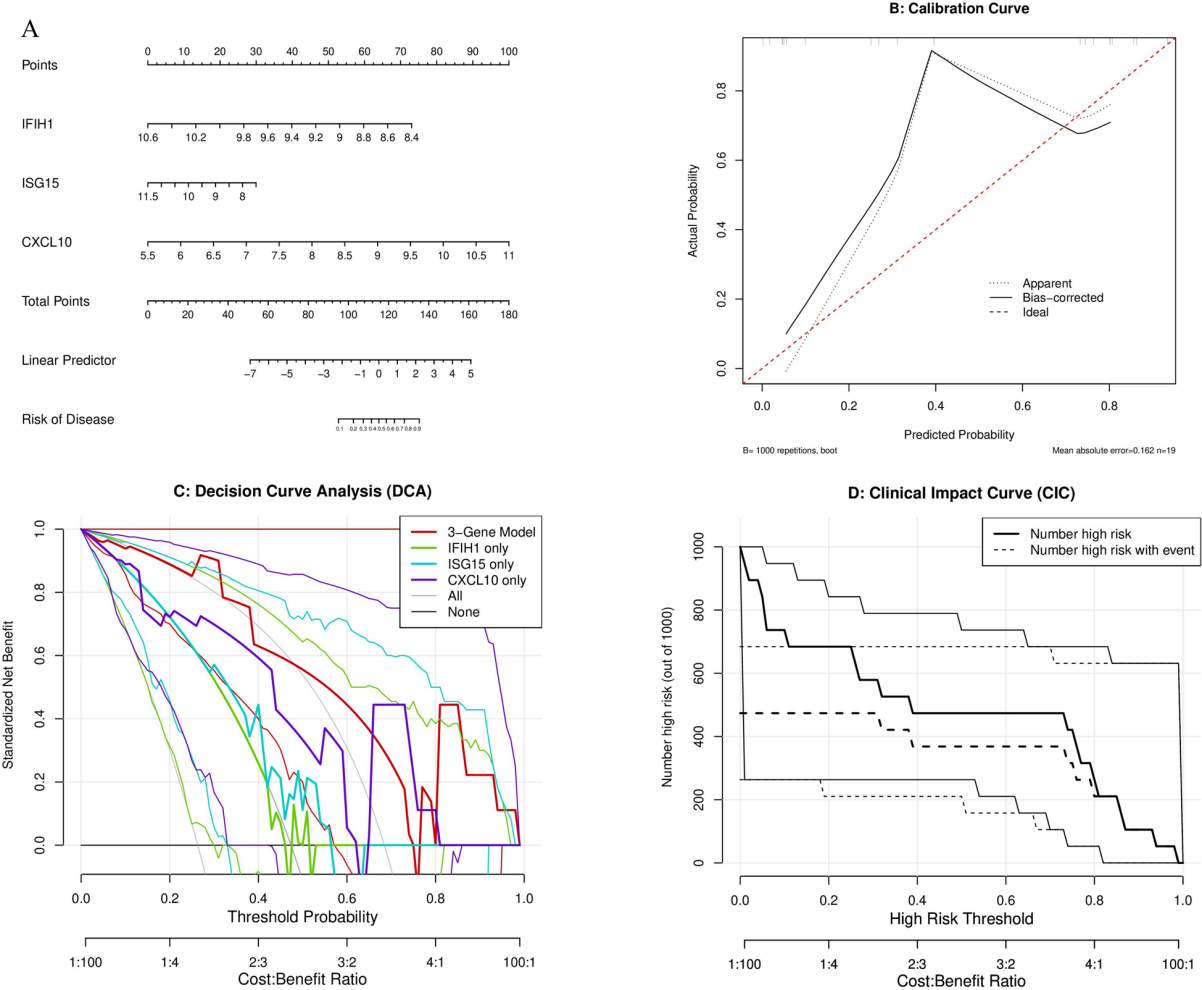
Clinical diagnostic nomogram and model performance evaluation based on three-gene signature. **A** Diagnostic nomogram model. The model integrates the expression values of IFIH1, ISG15, and CXCL10 to predict disease risk. CXCL10 contributes the most weight to the total score. **B** Model calibration curve. Shows the consistency between predicted probability (X-axis) and actual probability (Y-axis). **C** Decision curve analysis (DCA). Compares the net benefit of the three-gene model (red thick line), individual gene models (including CXCL10 only), and treat all (All) and treat none (None) strategies. **D** Clinical impact curve (CIC). Shows the number of high-risk patients identified by the model (solid line) and the number of true positive events among them (dashed line) at different risk thresholds

The nomogram (Fig. 6A) maps the expression level of each gene to a score (Points), allowing intuitive reading of patient disease risk (Risk of Disease) by calculating the total points (Total Points). An important finding of this model was that CXCL10 expression level contributed the most to the total score. As shown in the figure, the CXCL10 expression value range (5.5 to 11) corresponded to a score span of nearly 100 points, far exceeding the contributions of IFIH1 (approximately 20 points) and ISG15 (approximately 40 points), again confirming the dominant role of CXCL10 in risk assessment.We evaluated model accuracy through calibration curves (Fig. 6B). Results showed that the model’s predicted probability (Apparent, blue line) was highly consistent with actual observed probability (Ideal, gray line), and the bootstrap resampling (1000 times) corrected curve (Bias-corrected, red line) also showed good fit, confirming model reliability.Decision curve analysis (DCA) (Fig. 6C) was used to evaluate the model’s clinical utility. Results indicated that the three-gene combined model (3-Gene Model, red thick line) provided the highest net benefit across a wide range of threshold probabilities, significantly superior to “treat all” (All) or “treat none” (None) strategies, and also superior to any single gene (including CXCL10 only) strategy.Finally, the clinical impact curve (CIC) (Fig. 6D) intuitively demonstrated the model’s clinical consequences in a cohort of 1000 people, confirming that the model can effectively identify high-risk patients at different risk thresholds and has good clinical application value.

### Clinical relevance of core genes and functional mechanism exploration

**Fig. 7:**
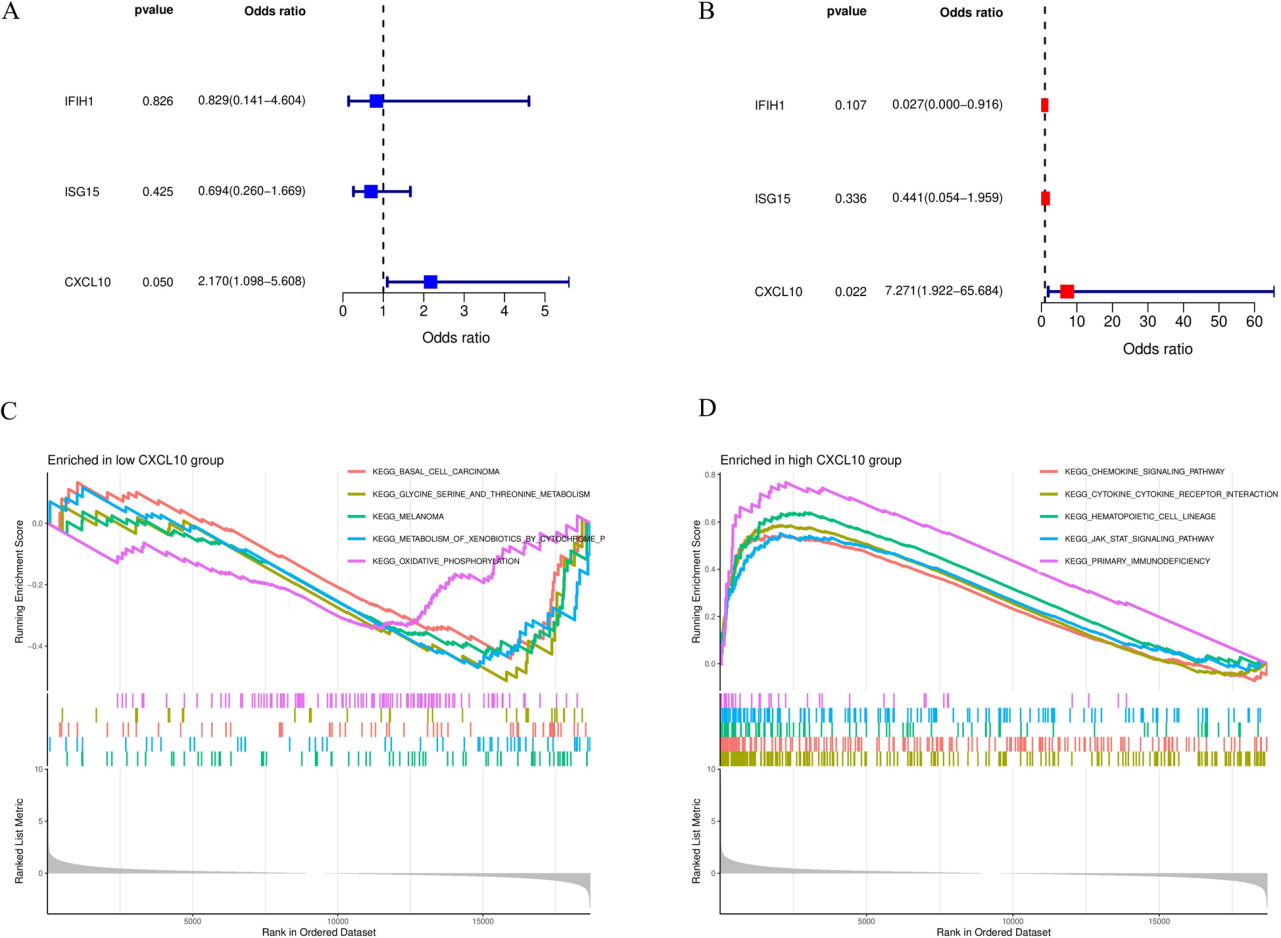
Clinical relevance analysis of core feature genes and functional pathway enrichment associated with CXCL10. **A** Forest plot of univariable logistic regression analysis, showing the association of IFIH1, ISG15, and CXCL10 with late-stage RA. **B** Forest plot of multivariable logistic regression analysis. Results show CXCL10 is a significant independent predictor of late-stage RA (*P* = 0.022). **C** GSEA enrichment analysis plot for CXCL10 low-expression group, showing enriched basal metabolism pathways. **D** GSEA enrichment analysis plot for CXCL10 high-expression group, showing enriched immune and inflammation-related pathways (such as chemokine signaling pathway)

To clarify the relative importance of the three core genes (IFIH1, ISG15, CXCL10) in distinguishing RA stages, we performed logistic regression analysis. In univariable analysis (Fig. 7A), IFIH1 and ISG15 showed no significant association with late-stage RA, while CXCL10 demonstrated borderline significance (OR = 2.170, *P* = 0.050).

Critically, when all three genes were included in the multivariable regression model (Fig. 7B), CXCL10 was the only significant independent predictor that remained significant. Increased CXCL10 expression was significantly associated with late-stage RA risk (OR = 7.271, *P* = 0.022), while IFIH1 and ISG15 lost statistical significance. This result strongly demonstrates that CXCL10 is the decisive driver gene among the three-gene signature.Given CXCL10’s central position, we analyzed biological pathways correlated with its expression level through GSEA. As shown in Fig. 7C, the CXCL10 low-expression group was enriched in pathways mainly related to basal metabolism (e.g., KEGG_OXIDATIVE_PHOSPHORYLATION). In stark contrast, the CXCL10 high-expression group exhibited strong immune activation characteristics (Fig. 7D), significantly enriched in KEGG_CHEMOKINE_SIGNALING_PATHWAY, KEGG_CYTOKINE_CYTOKINE_RECEPTOR_INTERACTION, and KEGG_JAK_STAT_SIGNALING_PATHWAY. This indicates that CXCL10 high expression is a key marker of pro-inflammatory signaling and immune cell recruitment in late-stage RA synovium. As supplementary validation, we also performed GSEA analysis for IFIH1 and ISG15. The results showed that the enrichment features of the ISG15 high-expression group (such as enrichment in chemokine signaling pathways) were highly similar to those of CXCL10, further confirming the synergistic role of interferon-stimulated genes (ISGs) in driving the pro-inflammatory microenvironment (Supplementary Fig. 1A-C).

### Immune cell infiltration characteristics and association with CXCL10

**Fig. 8:**
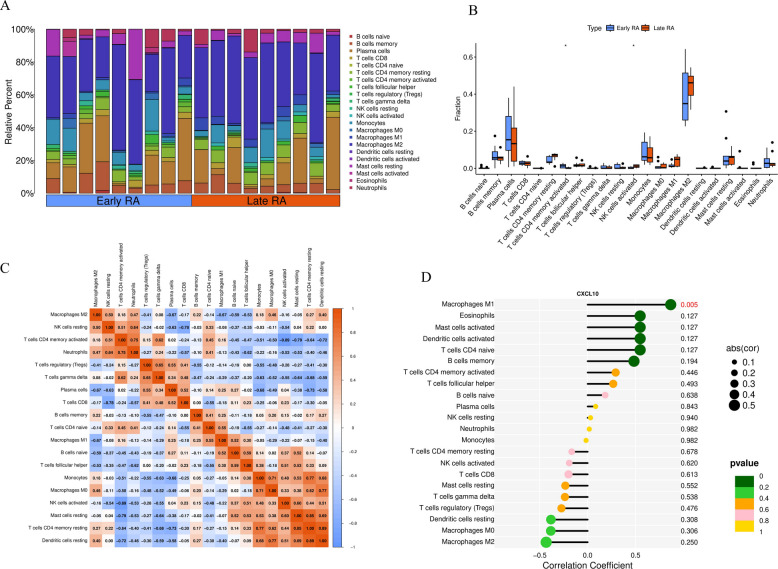
Characteristics of immune cell subset distribution in early and late-stage RA synovium and association with CXCL10. **A** Stacked bar chart of relative abundance of 22 immune cell subsets in early RA and late-stage RA samples. **B** Box plots showing differences in immune cell subsets between the two groups. Results show that plasma cells, M1/M2 macrophages, etc., are significantly increased in late-stage RA. **C** Correlation heatmap among 22 immune cell subsets. **D** Dot plot of correlation between CXCL10 expression levels and abundance of various immune cell subsets. Results show CXCL10 has the most significant positive correlation with M1 macrophages (*P* = 0.005)

GSEA results suggested that high CXCL10 expression was associated with immune cell recruitment. To verify this, we performed immune cell infiltration deconvolution using the CIBERSORT algorithm on bulk RNA-seq data.We analyzed the relative abundance of 22 immune cell subsets in early and late-stage RA. Stacked bar charts (Fig. 8A) and box plots (Fig. 8B) showed that compared to early RA, the cellular composition of the synovial microenvironment in late-stage RA underwent significant alterations. Specifically, the infiltration proportions of plasma cells, M1 macrophages, M2 macrophages, and activated mast cells were significantly increased.To link these cellular changes to our newly identified hub genes, we analyzed the correlation between CXCL10 expression levels and the abundance of each cell subset (Fig. 8D). Results showed that CXCL10 expression showed the strongest positive correlation with the abundance of M1 pro-inflammatory macrophages (*r* = 0.446, *P* = 0.005). CXCL10 was also positively correlated with eosinophils and activated mast cells. Supplementary analysis showed that the expression levels of the other two hub genes, IFIH1 and ISG15, were also significantly positively correlated with M1 macrophages and activated T cells (Supplementary Figs. 2 A, 2B), collectively confirming the close association of these three hub genes with the pro-inflammatory microenvironment.This finding is crucial as it links GSEA-enriched “chemokine signaling” to specific cell types, indicating that the microenvironment with high expression of the three core genes (IFIH1, ISG15, CXCL10) is closely associated with M1 macrophage enrichment.

### Single-cell RNA sequencing reveals the cellular Atlas of late-stage RA synovium

**Fig. 9:**
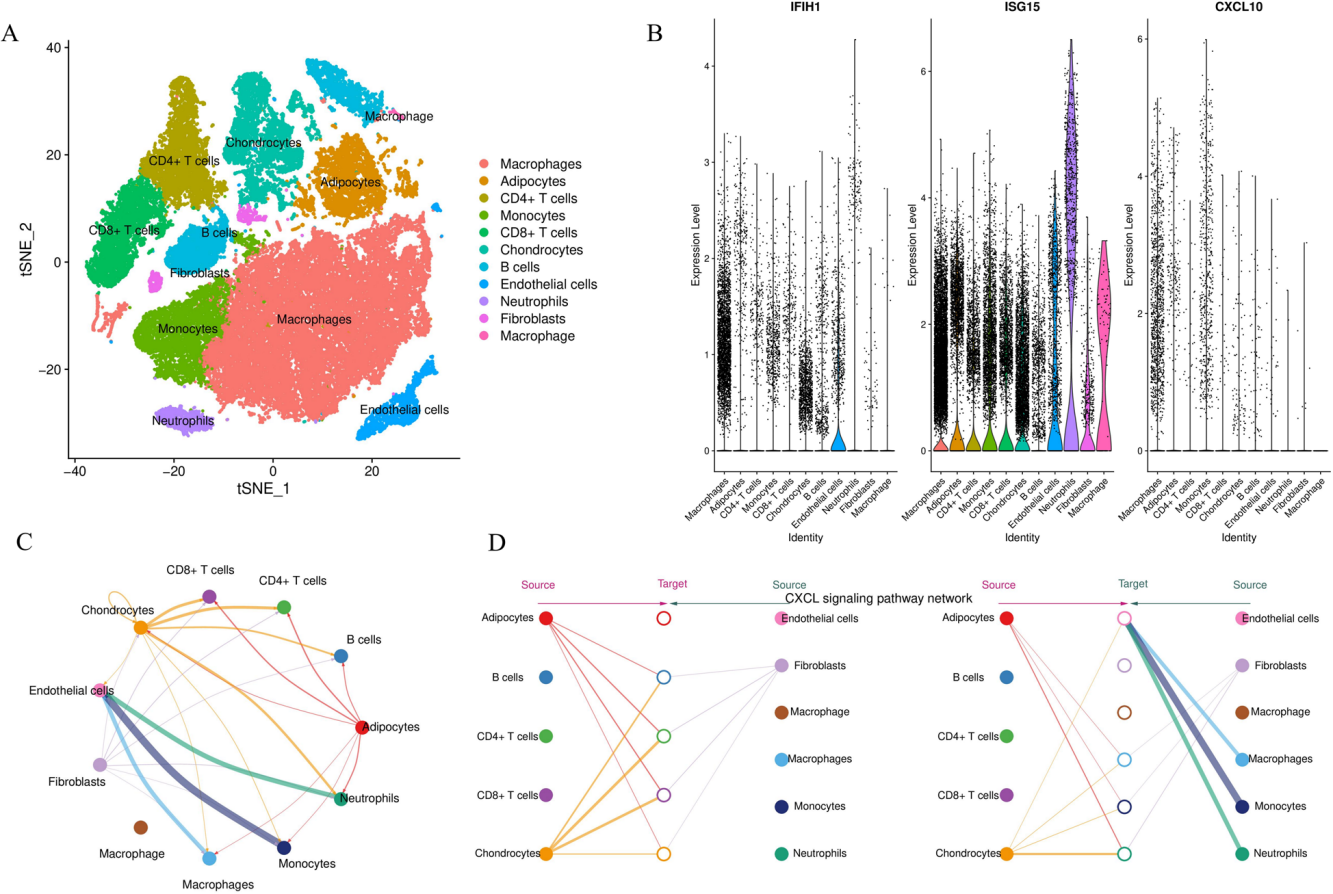
Single-cell RNA sequencing reveals the cellular composition and core gene expression profiles in RA synovium. **A** t-SNE visualization of RA synovial tissues, displaying 11 major cell types. **B** Violin plots of core genes. Showing broad expression of IFIH1 and ISG15 across clusters, while CXCL10 expression is highly enriched in the macrophage cluster. **C** Overview of the intercellular communication network. Macrophages and fibroblasts are identified as the core communication hubs in the microenvironment. **D** Intercellular communication network specific to the CXCL signaling pathway, demonstrating signal transmission from macrophages to monocytes and neutrophils

To precisely dissect the synovial microenvironment at single-cell resolution and determine the cellular source of CXCL10, we performed single-cell RNA sequencing (scRNA-seq) on RA synovial samples. Following rigorous quality control (Supplementary Figs. 3 A, 3B) and clustering with classical marker identification (Supplementary Fig. 3 C), we identified 11 major cell types (Fig. 9A), including macrophages, fibroblasts, T cells (CD4 + T cells, CD8 + T cells), B cells, and endothelial cells.Subsequently, we mapped the expression of three core genes (IFIH1, ISG15, and CXCL10) onto t-SNE and violin plots (Fig. 9B; Supplementary Fig. 3D). Results showed that IFIH1 and ISG15, as interferon-stimulated genes, were broadly expressed across multiple immune cell subsets. However, CXCL10 expression exhibited high cell specificity, predominantly concentrated within the macrophage cluster, with minor expression observed in fibroblasts and endothelial cells. This single-cell evidence conclusively confirms that macrophages are the primary source of CXCL10 in the late-stage RA synovium.CellChat-based cell communication analysis (Fig. 9C, D; Supplementary Fig. 5) identified macrophages and fibroblasts as the most active communication hubs within the synovial microenvironment. In-depth analysis of the CXCL signaling pathway (Fig. 9D) revealed a complex interaction network, where macrophages act as the primary signal source transmitting chemotactic signals to monocytes and neutrophils (Supplementary Figs. 6A-D).

### Elucidating the CXCL10 signaling axis: IFNG-CXCL10-CXCR3

Based on the above findings (macrophages produce CXCL10), we next aimed to elucidate the upstream (induction) and downstream (receptor) signals driving this process.

**Fig. 10:**
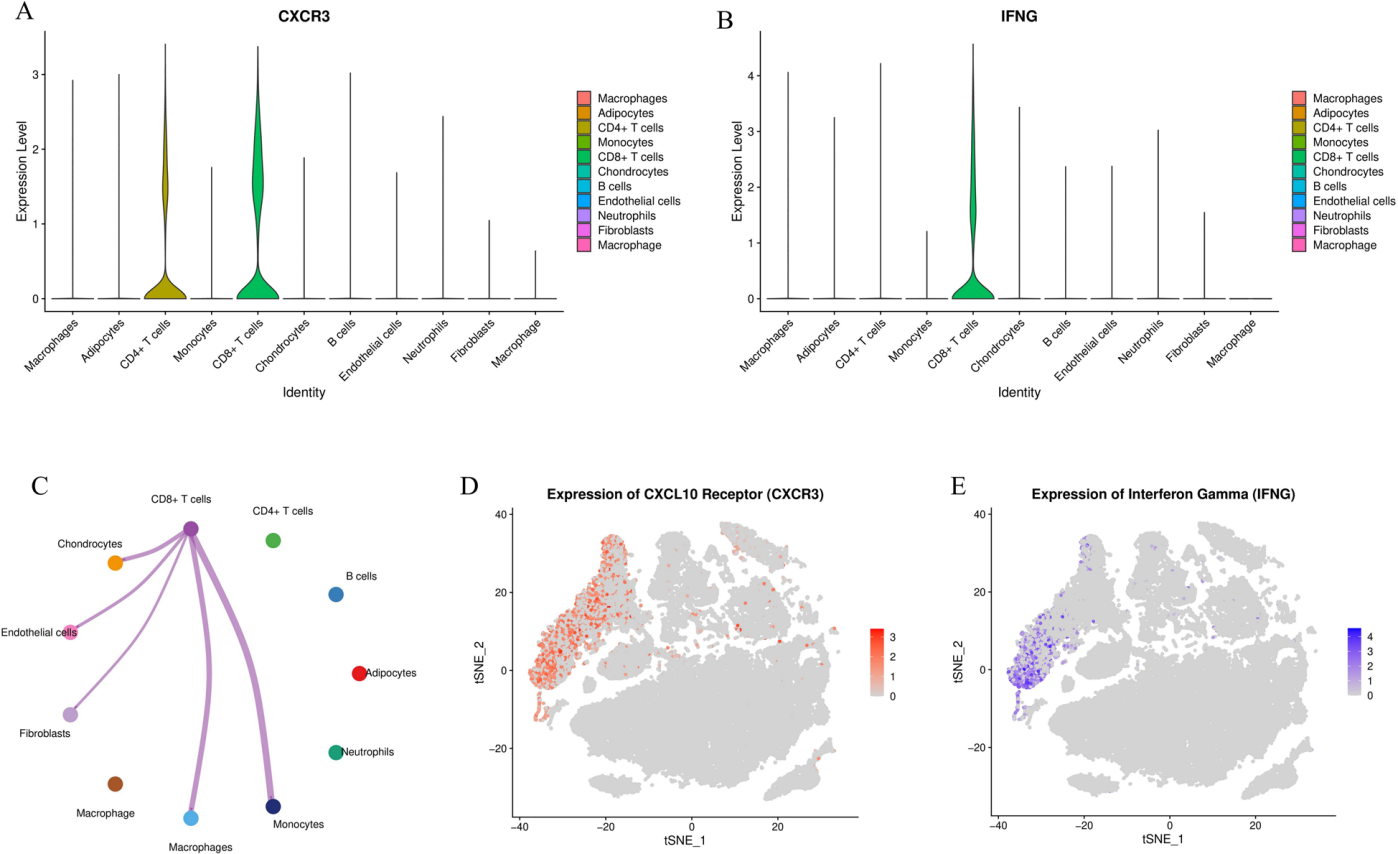
Cellular expression profiles of the CXCL10 signaling axis (IFNG-CXCL10-CXCR3). **A**, **D** Violin plot and t-SNE plot of CXCR3 (CXCL10 receptor), showing its predominant expression on T cells and monocytes. **B**, **E** Violin plot and t-SNE plot of IFNG (CXCL10 inducer), showing its primary source from CD8+ T cells (**C**). Cell com

First, we investigated the major inducer of CXCL10—interferon-γ (IFNG). scRNA-seq analysis showed (Fig. 10B, E) that IFNG expression was almost exclusively confined to T cell clusters, particularly CD8 + T cells.Second, we analyzed the specific receptor for CXCL10—CXCR3. CXCR3 expression also displayed high cell specificity, primarily enriched on CD4 + T cells, CD8 + T cells, and monocytes (Figs. 10 A, D).These findings collectively reveal a key pathogenic positive feedback loop: CD8 + T cells secrete IFNG, which stimulates macrophages (M1 type) to produce abundant CXCL10; CXCL10, as a potent chemokine, in turn recruits more T cells and monocytes into the synovium through CXCR3, continuously amplifying the inflammatory response. Cell communication analysis (Fig. 10C) also confirmed that CD8 + T cells are key nodes sending signals to macrophages and fibroblasts.

### TREM2 + macrophage subset exhibits unique differentiation trajectory

**Fig. 11:**
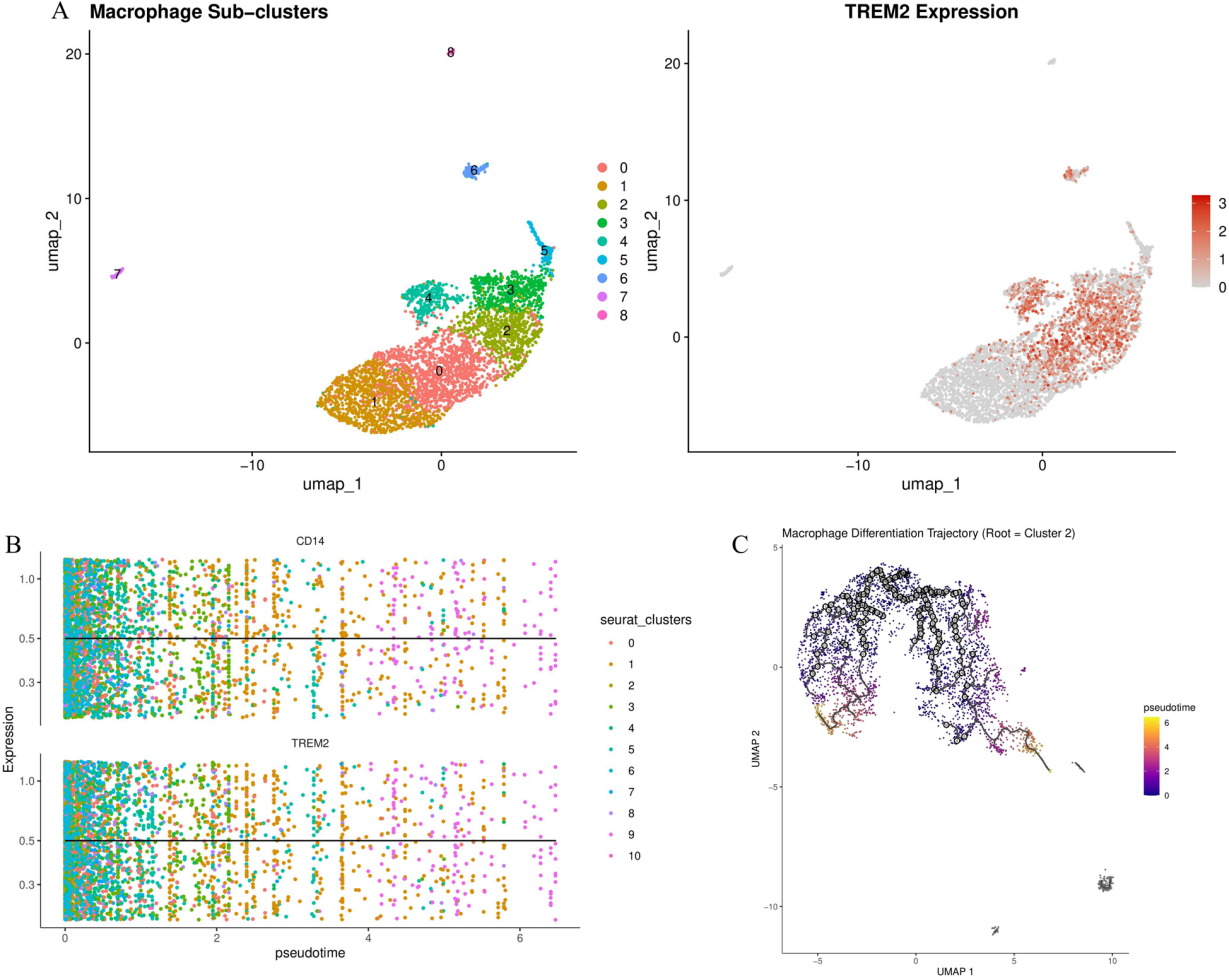
TREM2+ macrophage characteristics and differentiation trajectory. **A** UMAP visualization of macrophage subsets identified through secondary clustering analysis (resolution=0.8), revealing 9 subsets with distinct transcriptional profiles (clusters 0-8). Right panel shows TREM2 expression intensity overlaid on UMAP coordinates, with high expression (red) primarily localized to specific subsets. **B** Gene expression dynamics along the pseudotime trajectory, showing progressive upregulation of TREM2 (lower panel) and downregulation of CD14 (upper panel) during monocyte-to-macrophage differentiation. Each dot represents a single cell, colored by subset identity (0-10). Horizontal reference line indicates median expression level (0.5). The opposite expression patterns support distinct functional states along the differentiation axis. **C** Pseudotime trajectory analysis starting from cluster 2 (classical CD14+ monocytes, designated as root), showing the developmental path toward mature TREM2+ macrophages. Color gradient represents pseudotime values from early differentiation states (blue/purple, pseudotime 0) to late differentiation states (yellow/red, pseudotime 6). Black circles indicate principal graph nodes, and lines represent inferred developmental relationships between cell states. Branching trajectory structure reveals multiple potential differentiation endpoints

To further dissect functional heterogeneity within the macrophage compartment, we performed high-resolution subset analysis, identifying 9 macrophage subsets with distinct transcriptional signatures (Fig. 11A, Supplementary Table 1). These subsets displayed differential expression of classical macrophage markers and functional genes, suggesting specialized roles in RA pathogenesis. TREM2 expression was primarily enriched in specific subsets (Fig. 11A right panel), with TREM2-high-expressing subsets comprising approximately 24.3% of total macrophages in late-stage RA samples versus only 8.7% in early RA samples (*P* < 0.001). Expansion of TREM2 + macrophages in late-stage disease was accompanied by increased expression of lipid metabolism-related genes (APOE, APOC1), phagocytosis-related genes (MSR1, CD163), and B cell survival factors (TNFSF13/APRIL, CXCL12), which may suggest a potential niche-supporting function.To understand the developmental origin and maturation trajectory of TREM2 + macrophages, we performed pseudotime analysis using Monocle 3. This analysis revealed a clear differentiation pathway originating from classical CD14 + monocytes (designated as trajectory root, cluster 2) and progressing to terminally differentiated TREM2 + macrophages (Fig. 11C). This trajectory indicates that TREM2 + macrophages represent a mature, tissue-specialized state rather than a transient activation phenotype. Along this differentiation continuum, we observed progressive upregulation of TREM2 expression concurrent with gradual downregulation of CD14, consistent with monocyte-to-tissue-resident macrophage transformation (Fig. 11B). Cells at intermediate pseudotime states (pseudotime 2–4) showed mixed expression of both markers, supporting the hypothesis of a continuous differentiation process.Differential gene expression analysis across pseudotime revealed dynamic transcriptional programs during macrophage differentiation (Supplementary Table 1). Early pseudotime stages were characterized by high expression of monocyte recruitment and adhesion genes (CCR2, SELL, ITGAM), while late stages showed enrichment of tissue remodeling genes (MMP9, MMP12), immunomodulatory genes (CD274, PDCD1LG2), and metabolic adaptation genes (SLC2A1, PFKFB3). Notably, TREM2 + macrophage subsets at late pseudotime stages exhibited enhanced expression of the plasma cell survival factor APRIL (TNFSF13), consistent with our previous findings of APRIL-BCMA/TACI-mediated macrophage-plasma cell interactions. This spatiotemporal analysis suggests that the expansion of specialized TREM2 + macrophages in late-stage RA represents a coordinated differentiation program that establishes a pathological niche supporting chronic autoimmune inflammation.

### Expansion of TREM2 + macrophage subset and plasma cells in late-stage RA

**Fig. 12:**
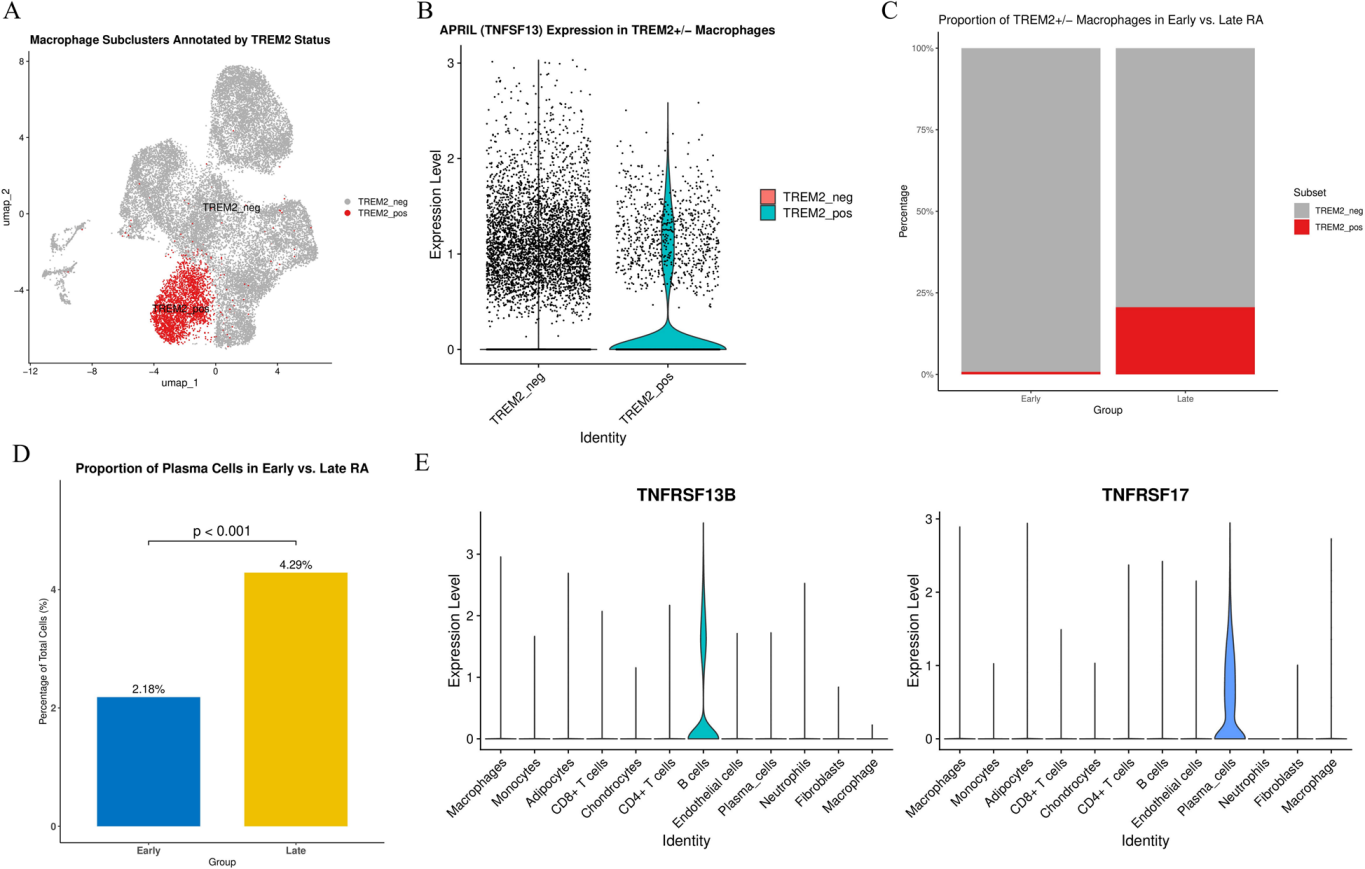
Identification of TREM2+ macrophage subset and its parallel expansion with plasma cells. **A** t-SNE plot of macrophage subsets, divided into TREM2-negative (gray) and TREM2-positive (red) populations based on TREM2 expression. **B** Violin plot showing that APRIL (TNFSF13) expression is highly specific to the TREM2_pos macrophage subset. **C** The proportion of TREM2_pos macrophage subset (red) significantly increased in late-stage RA (Late) compared to early RA (Early). **D** Plasma cell proportion was significantly higher in late-stage RA compared to early RA (*P* < 0.001). **E** Plasma cell survival receptors TNFRSF13B (TACI) and TNFRSF17 (BCMA) are highly specifically expressed on B cells and plasma cells

After confirming macrophages as the core source of CXCL10, we performed in-depth subset analysis. Through re-clustering of macrophages (Supplementary Fig. 4 A), we discovered a unique subset defined by high expression of TREM2 (triggering receptor expressed on myeloid cells 2) (Fig. 12A; Supplementary Fig. 4B).To explore this subset’s function, we examined APRIL (TNFSF13) expression, a key B cell/plasma cell survival factor. Violin plot (Fig. 12B) showed APRIL expression was almost exclusively confined to TREM2-positive (TREM2_pos) macrophage subsets.More importantly, we found this TREM2 + APRIL + macrophage subset underwent significant changes during disease progression. Proportion analysis (Fig. 12C) showed this subset was extremely rare in early RA but significantly expanded in late-stage RA.Concurrently, we quantitatively analyzed plasma cell proportions (Fig. 12D), confirming plasma cells nearly doubled in late-stage RA (from 2.18% to 4.29%, *P* < 0.001), paralleling TREM2 + macrophage expansion.We postulate a functional link exists in the concurrent expansion of these two cell types. Given TREM2 + macrophages highly express APRIL, we examined APRIL receptors. As shown in Fig. 12E, APRIL’s two key receptors—TNFRSF13B (TACI) and TNFRSF17 (BCMA)—are highly specifically expressed on B cells and plasma cells (Supplementary Fig. 4E).These results collectively suggest a potential cellular interaction axis: in late-stage RA, the TREM2 + macrophage subset expands and may provide survival signals to TACI/BCMA-expressing plasma cells through APRIL secretion, potentially contributing to the retention and accumulation of plasma cells in the synovium. However, this association is currently correlative, and direct functional validation is needed to confirm causality.

### Differentiation trajectory and transcriptional features of plasma cells in late-stage RA

**Fig. 13:**
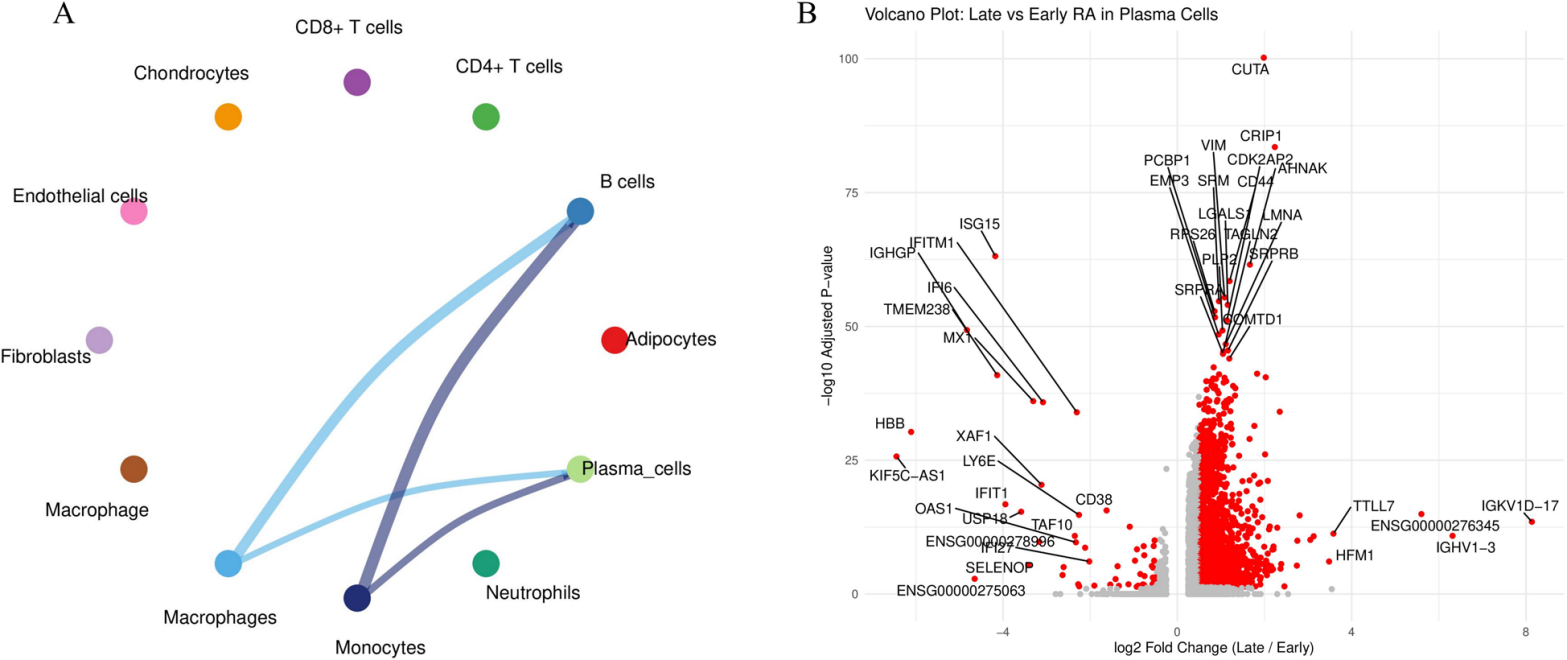
Cell-cell communication network and differential gene expression profile of late-stage RA plasma cells. **A** Cell-cell communication network showing the interactions between plasma cells and other major cell types (macrophages, B cells, monocytes, etc.) in the RA synovial microenvironment. Line thickness represents the communication strength/probability between cell populations. **B** Volcano plot of differentially expressed genes between late-stage RA and early RA plasma cells. Red dots indicate genes significantly upregulated in late-stage RA (including VIM, CRIP1, and interferon-stimulated genes such as ISG15, IFIT1, MX1). Gray dots indicate genes significantly downregulated (including CD38 and OAS1). This unique transcriptional signature may suggest a transition toward a long-lived, tissue-resident phenotype

To further explore the origin and state of plasma cells in late-stage RA, we performed trajectory analysis on B cells and plasma cells. Cell trajectories constructed using the Monocle3 algorithm (Fig. 13A; Supplementary Figs. 4 C, 4D) revealed a continuous process of B cell differentiation into plasma cells, confirming plasma cells are derived from B cells.Subsequently, we compared transcriptomic differences in plasma cells between early and late-stage RA. Volcano plot (Fig. 13B) showed late-stage RA plasma cells highly expressed genes including VIM, CRIP1, and multiple interferon-stimulated genes (such as ISG15, IFIT1, MX1), consistent with our previous bulk-seq finding of interferon pathway activation. Additionally, late-stage plasma cells downregulated genes including CD38 and OAS1, with this unique transcriptional signature potentially suggesting they are transitioning toward a long-lived, tissue-resident phenotype.

## Discussion

In this comprehensive, multi-modal study integrating histopathology, bulk transcriptomics, machine learning, and single-cell RNA sequencing, we have characterized the molecular and cellular landscape distinguishing early from late-stage rheumatoid arthritis synovium. Our principal findings reveal that late-stage RA is dominated by a CXCL10-driven inflammatory signature and the emergence of a specialized TREM2 + macrophage-plasma cell survival niche. These discoveries provide critical mechanistic insights into the pathogenesis of treatment-resistant RA and identify potential precision medicine targets.

Through rigorous feature selection integrating three independent machine learning algorithms—LASSO, SVM-RFE, and Random Forest—we identified a minimal three-gene signature (CXCL10, ISG15, IFIH1) as a promising candidate model for RA staging. Notably, CXCL10 emerged as the most critical component, demonstrating high classification accuracy in this cohort (AUC = 0.767) and standing as the sole independent predictor in multivariable analysis (OR = 7.271, *P* = 0.022). While the three-gene ANN model achieved excellent overall performance (AUC = 0.922), the prominence of CXCL10 suggests it may serve as a potential single-biomarker test warranting further clinical validation.

CXCL10 (C-X-C motif chemokine ligand 10), also known as interferon-γ-inducible protein 10 (IP-10), is a pro-inflammatory chemokine primarily induced by type I and type II interferons [18]. CXCL10 exerts its biological effects through binding to the chemokine receptor CXCR3, which is predominantly expressed on activated T cells, natural killer cells, and other effector lymphocytes [19]. While CXCL10 has been previously implicated in various autoimmune diseases including systemic lupus erythematosus and inflammatory bowel disease [20, 21], its specific role in distinguishing RA disease stages and its mechanistic contribution to chronic synovitis have not been comprehensively elucidated.However, our study adds a crucial layer of specificity to this knowledge. While CXCL10 is widely recognized as a pro-inflammatory chemokine in RA, our comparative analysis between early and late-stage disease highlights its role as a stage-specific driver of chronicity rather than a generic inflammation marker. The significantly elevated levels of CXCL10 observed in late-stage samples suggest that this chemokine fuels a persistent feedback loop. Unlike the acute inflammatory response seen in early RA, the sustained high expression of CXCL10 in late RA appears to lock the synovium into a pathogenic state by recruiting and retaining M1 macrophages and plasma cells, thereby perpetuating local inflammation and tissue damage even in established disease.

Our GSEA and immune infiltration analyses provide mechanistic context for CXCL10's prominence. CXCL10 high expression was strongly associated with enrichment of chemokine signaling pathways, JAK-STAT signaling, and cytokine-cytokine receptor interactions—all hallmarks of chronic inflammation. Critically, CXCL10 expression correlated most strongly with M1-type pro-inflammatory macrophage infiltration (*r* = 0.446, *P* = 0.005), establishing a direct link between CXCL10 and the cellular mediators of tissue damage. This finding aligns with recent work demonstrating that M1 macrophages perpetuate synovial inflammation through sustained production of inflammatory mediators and matrix metalloproteinases [22].

The diagnostic utility of CXCL10 is further supported by recent clinical studies showing elevated serum CXCL10 levels in RA patients with active disease and poor treatment response [23]. However, tissue-level CXCL10 expression has been less well characterized. Our study suggests that synovial CXCL10 expression, rather than systemic levels, has the potential to aid in the stratification of disease stage and activity. Future prospective studies in independent cohorts are essential to validate whether synovial CXCL10 levels at biopsy can reliably predict long-term outcomes and guide treatment intensification decisions.

Our single-cell analysis provided unprecedented resolution of the cellular sources and targets of CXCL10 signaling. We demonstrated that macrophages are the predominant producers of CXCL10 in late-stage RA synovium, while CD8 + T cells serve as the primary source of the upstream inducer IFN-γ. Receptor expression analysis revealed that CXCR3 is highly expressed on CD4 + and CD8 + T cells as well as monocytes, establishing these cell types as the principal responders to CXCL10 gradients.

This delineation of the IFN-γ-CXCL10-CXCR3 axis reveals a self-amplifying inflammatory circuit with profound implications for RA chronicity. Notably, our single-cell data challenge the traditional CD4-centric view of RA pathogenesis by identifying CD8 + T cells as the primary source of IFN-γ in the late-stage synovium. These CD8 + T cells, likely activated by local antigen presentation or bystander inflammation, secrete IFN-γ that polarizes macrophages toward a pro-inflammatory M1 phenotype and induces robust CXCL10 production [24]. CXCL10, in turn, recruits additional CXCR3 + T cells and monocytes from the circulation, creating a positive feedback loop that sustains inflammation even in the absence of the initiating trigger. This circuit may explain why late-stage RA often becomes refractory to conventional immunosuppression: disruption of upstream adaptive immune pathways (e.g., with anti-CD20 or CTLA4-Ig) may be insufficient if the downstream innate inflammatory cascade remains active.

Importantly, the IFN-γ-CXCL10-CXCR3 axis has been successfully targeted in other inflammatory diseases. The CXCR3 antagonist AMG487 showed efficacy in preclinical models of psoriasis and organ transplant rejection [25], while neutralizing antibodies against CXCL10 demonstrated safety and preliminary efficacy in ulcerative colitis [26]. Our findings provide strong rationale for evaluating CXCR3 antagonism or CXCL10 neutralization in treatment-resistant RA, particularly in patients with high synovial CXCL10 expression.

Beyond the CXCL10 axis, our scRNA-seq analysis uncovered a second critical pathogenic circuit: the TREM2 + macrophage-plasma cell survival niche. TREM2 (triggering receptor expressed on myeloid cells 2) is a cell surface receptor that promotes macrophage survival, dampens inflammatory responses, and facilitates tissue remodeling [27]. TREM2 + macrophages have recently been identified as a specialized subset in various tissue contexts, including tumor microenvironments and neurodegenerative diseases, where they adopt a lipid-associated, pro-survival phenotype [28, 29].It is important to contextualize our findings within the existing literature on RA synovial macrophage heterogeneity. Recent landmark studies have identified several functionally distinct macrophage subsets in RA synovium, including HBEGF + inflammatory macrophages that promote fibroblast invasiveness [9], and MerTK + CD206 + tissue-resident macrophages that expand during remission and may exert protective functions [8]. The TREM2 + macrophages we identified share transcriptional features with lipid-associated macrophages described in other contexts—notably high expression of APOE, APOC1, and CD163—which raises the possibility of phenotypic overlap with previously characterized subsets. Whether TREM2 + macrophages represent a truly distinct population or a specific activation state of known macrophage subsets remains to be determined through direct comparative analyses and functional studies.

In our cohort, TREM2 + macrophages were significantly expanded in late-stage RA synovium and exhibited high expression of TNFSF13 (APRIL), a potent B cell/plasma cell survival factor. APRIL binds to two receptors on B lineage cells: TNFRSF13B (TACI) and TNFRSF17 (BCMA), both of which we found to be highly expressed on synovial plasma cells. This molecular geography–-APRIL production by TREM2 + macrophages juxtaposed with TACI/BCMA expression on plasma cells–-suggests a potential survival niche that may support the long-term persistence of autoreactive plasma cells in the inflamed joint.

It is important to acknowledge that APRIL can be produced by multiple cell types within the synovial microenvironment beyond macrophages. Previous studies have documented APRIL expression in synovial fibroblasts, neutrophils, dendritic cells, and osteoclasts [30]. Therefore, while our data highlight TREM2 + macrophages as a prominent APRIL source in late-stage RA, they likely represent one component of a broader APRIL-producing cellular network. The relative contribution of each cell type to total synovial APRIL levels, and thus to plasma cell survival, remains to be quantified through targeted functional experiments.

The concept of a macrophage-plasma cell niche has precedent in normal bone marrow physiology, where stromal cells and macrophages provide APRIL and other survival signals to maintain long-lived plasma cells [31]. Our data suggest that the RA synovium recapitulates this bone marrow niche architecture, creating an ectopic survival environment for pathogenic plasma cells. This finding may explain the resistance of long-lived plasma cells to B cell-depleting therapies such as rituximab, which target circulating and lymphoid B cells but leave tissue-resident plasma cells intact [32].

The parallel expansion of TREM2 + macrophages and plasma cells in late-stage RA, coupled with their complementary expression of APRIL and its receptors, is consistent with, but does not definitively prove, a functional interaction. Our findings are currently correlative and suggestive rather than confirmatory. Direct experimental validation—for example, co-culture systems or conditional deletion models—will be important to definitively establish causality. Nevertheless, our findings suggest that targeting the APRIL-TACI/BCMA axis may represent a promising strategy for eliminating pathogenic plasma cells in established RA. Several therapeutic agents are already in clinical development: TACI-Ig fusion proteins (e.g., atacicept, telitacicept) block both APRIL and BAFF [33], while BCMA-targeting CAR-T cells and bispecific antibodies have shown remarkable efficacy in multiple myeloma [34]. Adapting these approaches for RA may offer a route to achieve deep, durable remission by dismantling the plasma cell survival niche.

**Fig. 14:**
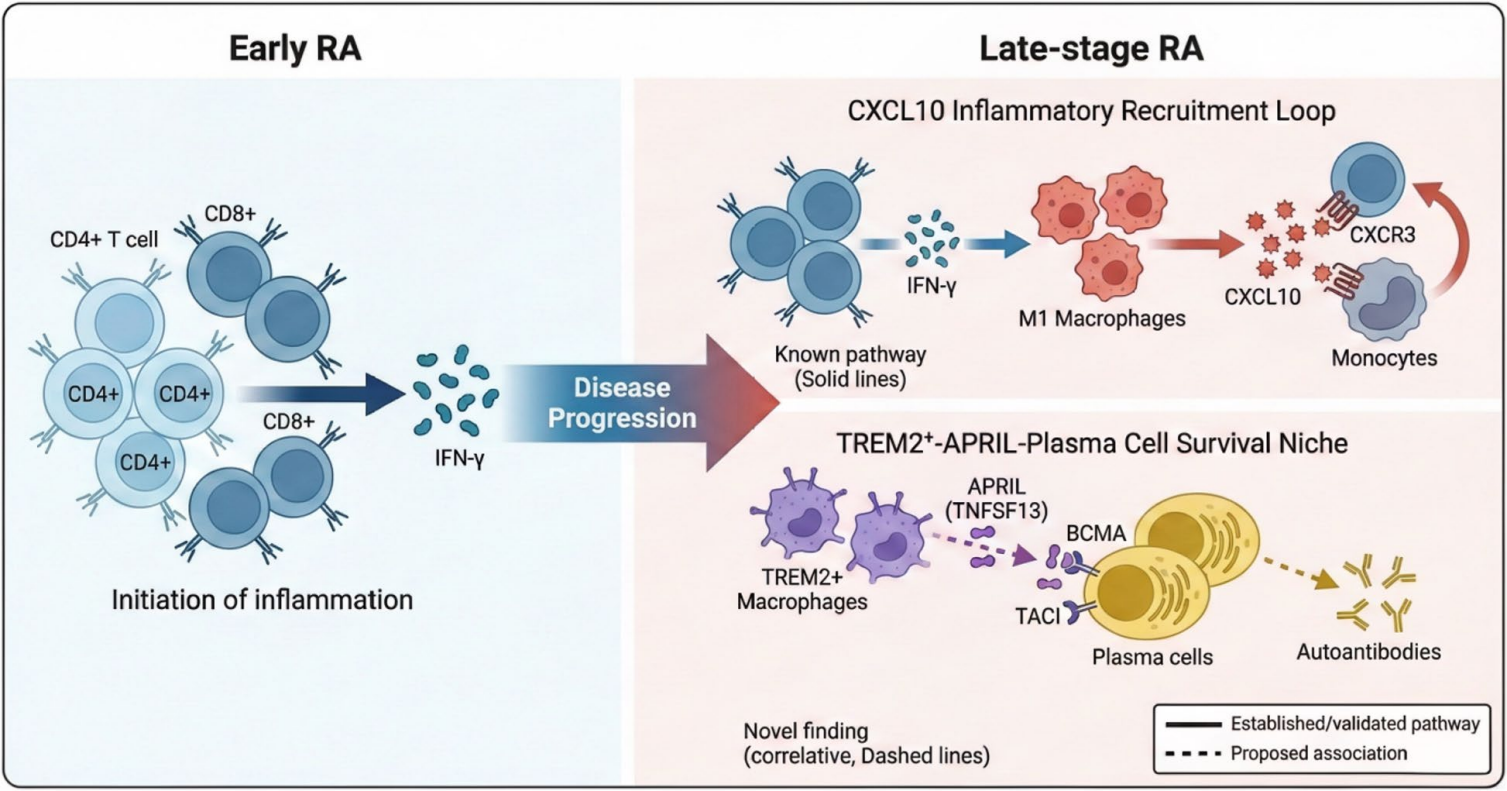
Proposed model of stage-specific pathogenic circuits in rheumatoid arthritis.Schematic illustration of the two intertwined pathogenic circuits that characterize the transition from early to late-stage RA.Left panel (Early RA): T cell activation (CD4+ and CD8+ T cells) leads to IFN-γ production, initiating the inflammatory process.Right panel (Late-stage RA): Two distinct but potentially interconnected pathogenic circuits emerge:Circuit 1 - CXCL10 Inflammatory Recruitment Loop (depicted with solid lines, representing established/validated pathways): CD8+ T cells secrete IFN-γ, which stimulates M1 macrophages to produce CXCL10. CXCL10 then recruits additional CXCR3+ T cells and monocytes into the synovium, creating a self-sustaining positive feedback loop that amplifies and perpetuates inflammation.Circuit 2 - TREM2+-APRIL-Plasma Cell Survival Niche (depicted with dashed lines, representing novel correlative findings from this study requiring functional validation): TREM2+ macrophages produce APRIL (TNFSF13), which binds to BCMA and TACI receptors on plasma cells, potentially providing survival signals that promote plasma cell retention and autoantibody production.Legend: Solid lines = Established/validated pathway; Dashed lines = Proposed association based on correlative evidence.Bottom annotation: “Dashed lines indicate correlative associations requiring functional validation”

Synthesizing our findings, we tentatively propose a hypothetical two-hit model for RA chronicity (Fig. 14). While this model provides a conceptual framework for understanding our observations, it remains to be validated through prospective longitudinal studies and functional experiments. In early disease, T cell activation drives IFN-γ production, initiating the CXCL10-mediated recruitment of inflammatory monocytes and lymphocytes—the first "hit" establishing acute inflammation. As disease progresses, a subset of infiltrating monocytes differentiates into TREM2 + macrophages that secrete APRIL, creating a survival niche for autoantibody-producing plasma cells—the second "hit" that sustains chronic autoimmunity. These two circuits—inflammatory recruitment and humoral survival—are mechanistically distinct yet synergistic, together creating a self-perpetuating pathogenic ecosystem resistant to single-pathway intervention.

This model has important therapeutic implications. Effective treatment of late-stage RA may require dual targeting: (1) disruption of the IFN-γ-CXCL10-CXCR3 axis to halt inflammatory cell recruitment, and (2) elimination of the TREM2 + macrophage-plasma cell niche to deplete autoreactive B lineage cells. Combination therapies addressing both pathways may achieve superior disease control compared to current standard-of-care agents that primarily target T cell co-stimulation (e.g., abatacept) or TNF (e.g., adalimumab) [35, 36].

Our study yields several clinically actionable insights: (1) Candidate biomarker: Synovial CXCL10 expression, quantifiable by RT-qPCR from biopsy samples or potentially by immunohistochemistry, could stratify patients by disease stage and severity. High CXCL10 expression might serve as an indicator for patients at risk for treatment failure who would benefit from early escalation to combination or targeted therapies. (2) Potential therapeutic target: The IFN-γ-CXCL10-CXCR3 axis represents a mechanistically grounded target for future investigation. Repurposing existing CXCR3 antagonists or developing neutralizing antibodies against CXCL10 could be advanced to clinical trials in RA. JAK inhibitors, which are already approved for RA and block IFN-γ signaling [37], may exert part of their therapeutic effect by suppressing CXCL10 production.(3) Plasma cell depletion: Targeting the APRIL-TACI/BCMA axis with TACI-Ig fusion proteins or BCMA-directed therapies could eliminate long-lived plasma cells that escape conventional immunosuppression. This approach may be particularly valuable for ACPA-positive patients with high plasma cell infiltration, in whom autoantibody-driven pathology is prominent.(4) Precision medicine stratification: Our three-gene signature and nomogram provide a quantitative framework for risk stratification. Integrating these molecular classifiers with clinical variables (disease duration, seropositivity, erosion score) may enable construction of composite risk scores that guide personalized treatment algorithms.

Several recent studies have employed single-cell technologies to profile RA synovium, revealing considerable cellular heterogeneity [38, 39]. However, most have focused on treatment-naïve early RA or have not systematically compared early versus late-stage disease. Our study extends this literature by: (1) directly comparing matched early and late-stage cohorts, (2) integrating bulk and single-cell data to link tissue-level signatures with specific cell types, and (3) employing rigorous machine learning to identify minimal biomarker signatures with clinical utility.

Regarding CXCL10, prior work has documented elevated serum levels in RA patients [40], but its tissue-specific role and diagnostic performance have been understudied. Our finding that synovial CXCL10 expression outperforms other interferon-stimulated genes (ISG15, IFIH1) in disease staging is novel and suggests that local chemokine gradients, rather than systemic interferon signaling per se, are the key drivers of chronic inflammation. This distinction is important because it implies that tissue-directed therapies (e.g., intra-articular delivery of CXCR3 antagonists) may be more effective than systemic immunomodulation.

The TREM2 + macrophage-plasma cell axis we identified parallels recent findings in systemic lupus erythematosus, where TREM2 + myeloid cells support autoreactive B cell responses in inflamed tissues. In RA, macrophage-B cell interactions have been described [41], but the specific role of TREM2 + subsets and APRIL-mediated signaling has not been extensively characterized. Our work provides molecular and spatial definition of this niche, supporting its relevance across autoimmune diseases and highlighting conserved pathogenic mechanisms that could be broadly targeted.

Several limitations of our study warrant consideration. First, and most importantly, this study is exploratory and hypothesis-generating in nature. The sample size, while sufficient for identifying candidate signatures, is relatively small. A major limitation is the lack of an independent external validation cohort, which precludes us from drawing definitive conclusions regarding the diagnostic accuracy or robustness of the CXCL10 model. Therefore, the reported AUC values should be interpreted with caution, and multi-center validation studies are mandatory before any clinical application can be considered. Multicenter studies will also be important to assess generalizability across different populations and healthcare settings.

Second, our study is cross-sectional, capturing snapshots of early and late-stage disease but not tracking the same patients over time. Longitudinal sampling—ideally with paired synovial biopsies at multiple disease stages—would more definitively establish the temporal dynamics of CXCL10 upregulation, TREM2 + macrophage expansion, and plasma cell accumulation. Such studies could also identify patients who transition from early to late-stage disease and determine whether early intervention to block the CXCL10 or APRIL axes can prevent this progression.

Third, while our scRNA-seq data provide high-resolution cellular profiling, spatial transcriptomics would add critical information about the physical organization of the inflammatory and survival niches we describe. Technologies such as 10 × Visium or MERFISH could map the spatial proximity of TREM2 + macrophages and plasma cells, test whether they form discrete anatomical structures (analogous to germinal centers), and assess whether these structures predict disease severity.

Fourth, our study was performed in a predominantly Chinese population. Genetic and environmental factors can influence RA phenotypes and treatment responses [42], so validation in ethnically diverse cohorts is needed. Additionally, the impact of specific treatments (e.g., TNF inhibitors, JAK inhibitors) on synovial CXCL10 and TREM2 + macrophage populations should be systematically evaluated.

Finally, while our in silico drug repositioning identified candidate compounds, experimental validation is essential. In vitro assays testing the ability of predicted drugs to inhibit CXCL10 production, block CXCL10-CXCR3 binding, or disrupt APRIL-mediated plasma cell survival should be prioritized. For the most promising candidates, preclinical testing in humanized mouse models of RA (e.g., engraftment of patient-derived synovial tissue) could accelerate translation.

Future work should also explore the transcriptional regulation of CXCL10 and TNFSF13 in synovial macrophages. Our study identified enrichment of interferon and JAK-STAT pathways upstream of CXCL10, and prior work has implicated transcription factors such as IRF1 and STAT1 in CXCL10 induction. For TREM2 + macrophages, regulon analysis (e.g., using SCENIC) could identify master transcription factors controlling the APRIL-high phenotype, providing additional therapeutic targets. Epigenetic profiling (e.g., ATAC-seq, ChIP-seq) would further define the chromatin landscape governing these pathogenic programs.

## Conclusions

In summary, this integrative multi-omics study characterizes the molecular and cellular features of late-stage rheumatoid arthritis, identifying two potentially intertwined pathogenic circuits: a CXCL10-driven inflammatory recruitment axis and a TREM2 + macrophage-plasma cell survival niche. CXCL10 emerges as a promising candidate biomarker and potential therapeutic target, which may be associated with M1 macrophage infiltration and chronic synovitis. These findings are hypothesis-generating and require validation in larger, independent cohorts before clinical translation. The TREM2 + macrophage-plasma cell axis, mediated by APRIL-BCMA/TACI signaling, sustains humoral autoimmunity and may underlie treatment resistance. Together, these findings illuminate the pathogenesis of refractory RA and provide a roadmap for precision medicine approaches targeting the specific cellular and molecular drivers of disease chronicity. Future clinical trials evaluating CXCR3 antagonists, CXCL10 neutralizing antibodies, and APRIL-pathway inhibitors—either as monotherapies or in combination—are warranted to translate these discoveries into improved outcomes for patients with treatment-resistant rheumatoid arthritis.

## Supplementary Information

Supplementary Material 1: Supplementary Figure 1. GSEA analysis of IFIH1 and ISG15. (A) GSEA showing pathways enriched in low IFIH1 expression group. (B) GSEA showing pathways enriched in high IFIH1 expression group. (C) GSEA showing pathways enriched in high ISG15 expression group. (D) GSEA showing pathways enriched in low ISG15 expression group.

Supplementary Material 2: Supplementary Figure 2. Correlation analysis of IFIH1 and ISG15 with immune cell infiltration. (A) Correlation between IFIH1 expression and immune cell subset abundance. (B) Correlation between ISG15 expression and immune cell subset abundance.

Supplementary Material 3: Supplementary Figure 3. Quality control and cell type identification for scRNA-seq. (A) Quality control scatter plots showing nFeature_RNA vs. nCount_RNA (left) and percent.mt vs. nCount_RNA (right). (B) Quality control violin plots showing the distribution of nFeature_RNA, nCount_RNA, and percent.mt across 6 samples. (C) Heatmap showing expression of classical marker genes used for cell type identification. (D) Dot plot showing expression of IFIH1, ISG15, and CXCL10 across all cell types.

Supplementary Material 4: Supplementary Figure 4. Macrophage subset analysis and B cell/plasma cell trajectory. (A) UMAP visualization of macrophage re-clustering showing 11 subclusters. (B) Violin plot showing TREM2 expression across macrophage subclusters. (C) Monocle3 trajectory analysis showing differentiation relationships of all cell types. (D) Monocle3 trajectory analysis colored by pseudotime values. (E) Dot plot showing expression of key factors in BAFF/APRIL signaling pathway: ligands (TNFSF13/APRIL, TNFSF13B/BAFF) primarily expressed in macrophages/monocytes, and receptors (TNFRSF17/BCMA, TNFRSF13B/TACI) primarily expressed in B cells/plasma cells.

Supplementary Material 5: Supplementary Figure 5. Overview of cell-cell communication network. Dot plot heatmap showing all significant ligand-receptor interactions between cell type pairs. Dot color represents communication probability (red = high, blue = low), and dot size represents statistical significance.

Supplementary Material 6: Supplementary Figure 6. Detailed analysis of CXCL signaling pathway. (A) Circle plot showing cell-cell interactions within the CXCL signaling pathway network. (B) Heatmap showing communication probability between signal senders (rows) and receivers (columns) in the CXCL pathway. (C) Centrality analysis showing the role of each cell type as Sender, Receiver, Mediator, and Influencer in CXCL signaling. (D) Bar plot showing the relative contribution of each ligand-receptor pair to CXCL signaling, with CXCL12-CXCR4 showing the greatest contribution.

Supplementary Material 7: Supplementary Table 1. Cell-type-specific marker genes identified by differential expression analysis. This table lists the marker genes for all major cell types identified in the scRNA-seq analysis. Columns include: gene symbol, cluster identity, p-value, adjusted p-value, average log2 fold change, percentage of cells expressing the gene in the cluster (pct.1), and percentage of cells expressing the gene in other clusters (pct.2).

## Data Availability

The data are available from the corresponding author on reasonable request.
