# Supplementary figures and images for: Integrative multi-omics analysis reveals CXCL10-driven inflammation and TREM2 + macrophage-plasma cell survival niche as hallmarks of late-stage rheumatoid arthritis
Source: Arthritis Res Ther. 2026 Feb 12;28:70. doi: 10.1186/s13075-026-03764-3 (PMC12997883; doi:10.1186/s13075-026-03764-3)

A

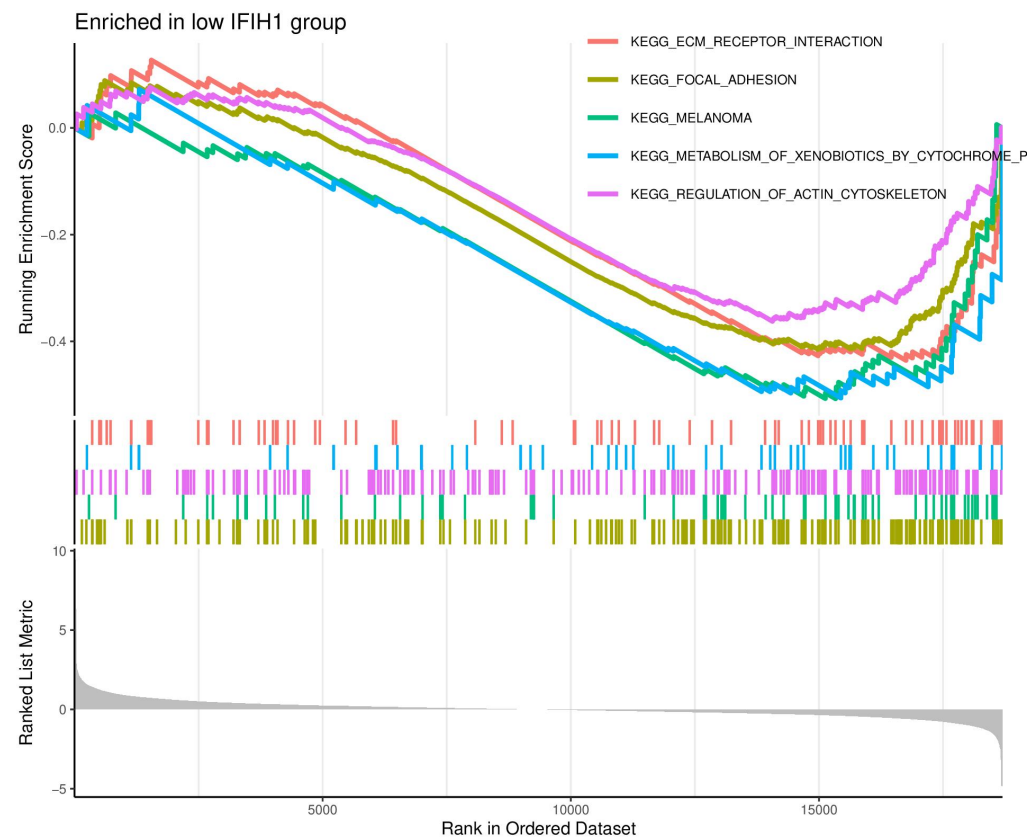

B

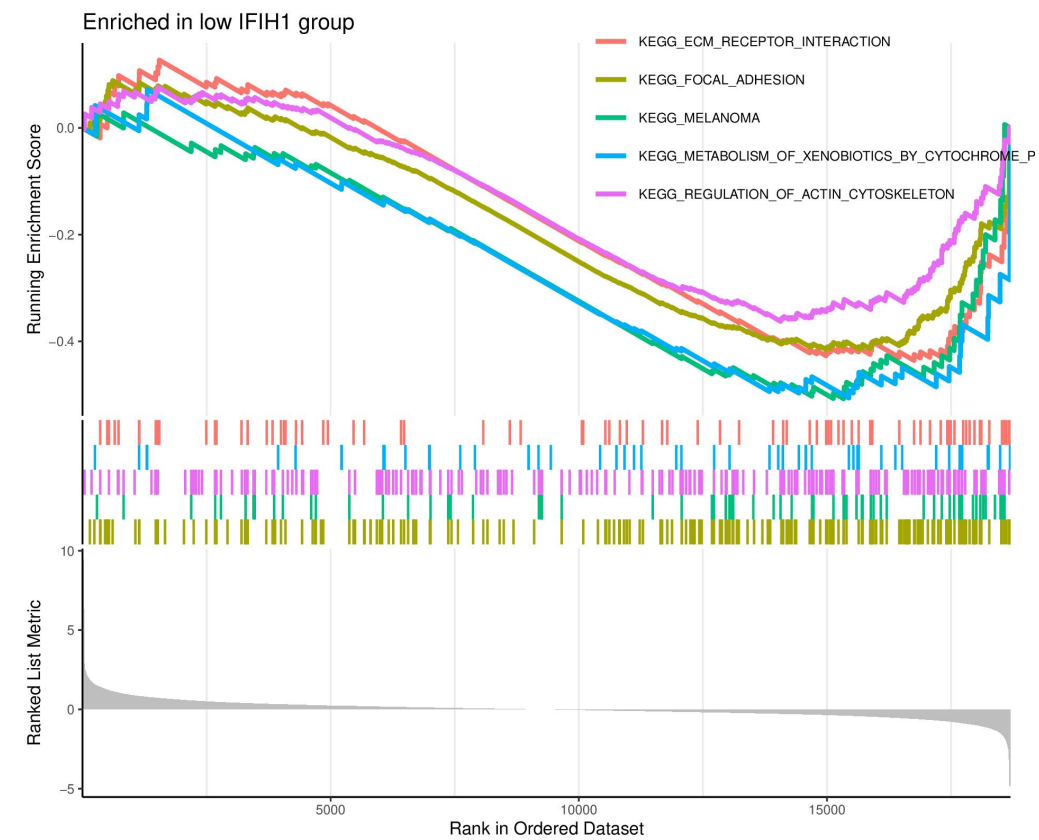

C

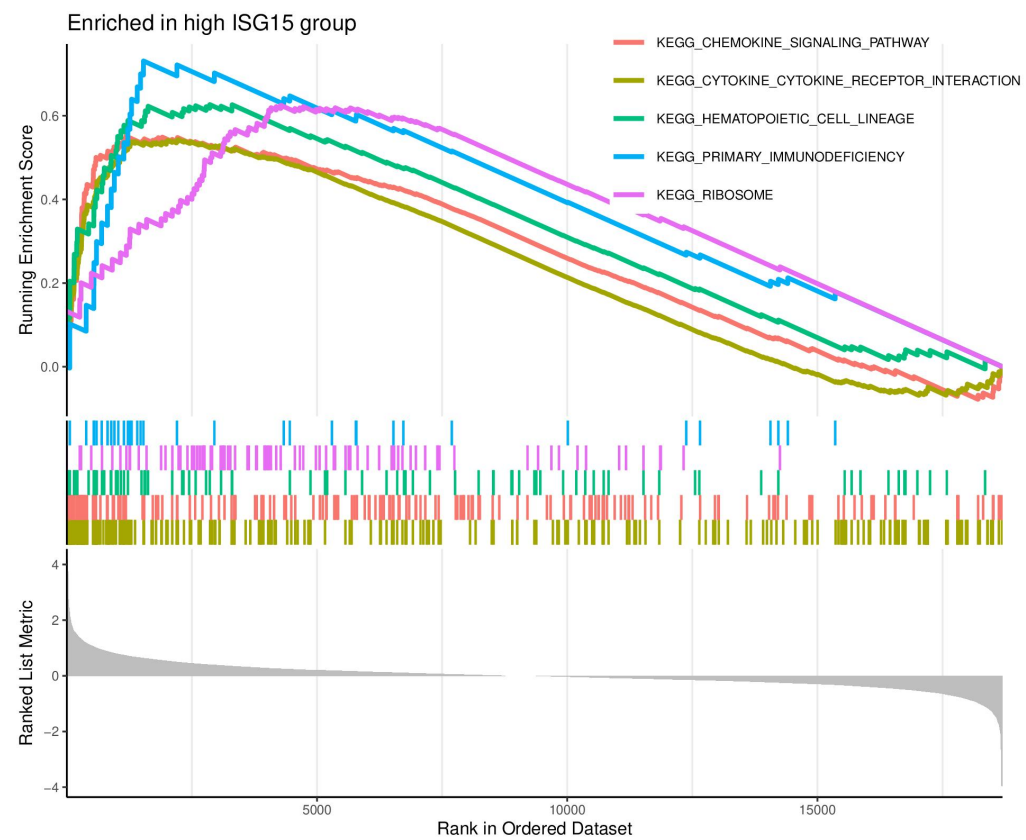

D

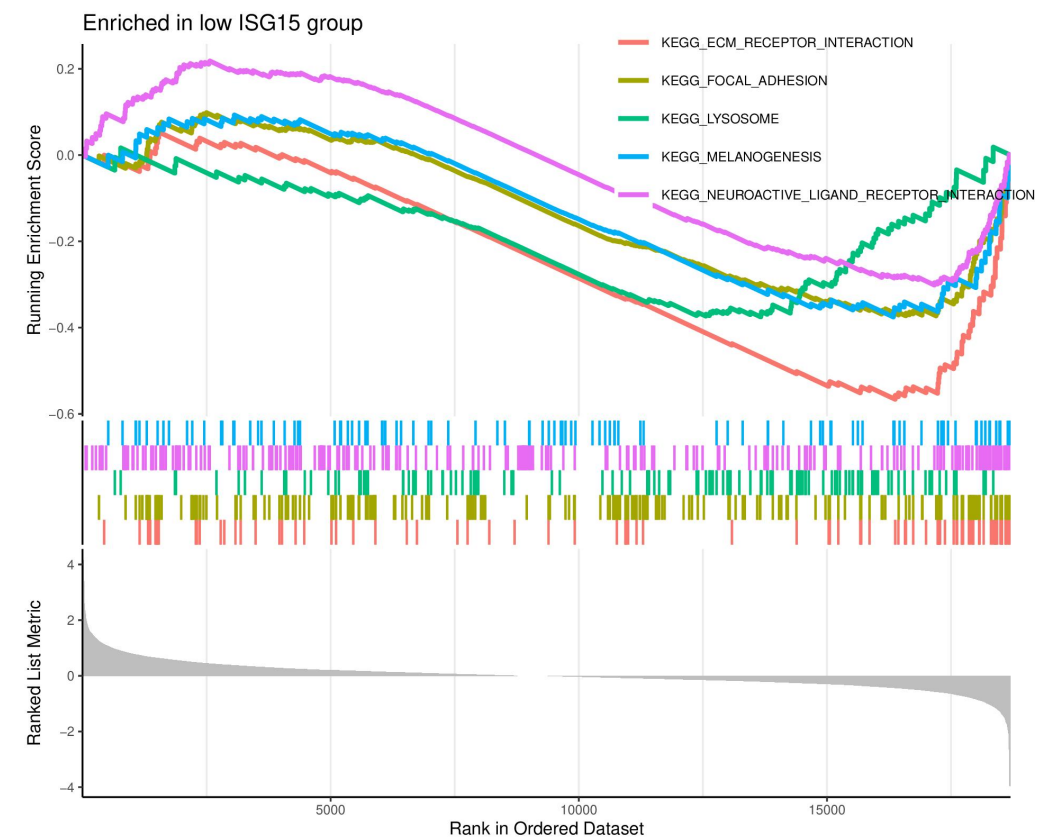

Supplement: Supplementary file 1 — Supplementary Material 1: Supplementary Figure 1. GSEA analysis of IFIH1 and ISG15. (A) GSEA showing pathways enriched in low IFIH1 expression group. (B) GSEA showing pathways enriched in high IFIH1 expression group. (C) GSEA showing pathways enriched in high ISG15 expression group. (D) GSEA showing pathways enriched in low ISG15 expression group. [file 13075_2026_3764_MOESM1_ESM.pdf]

A

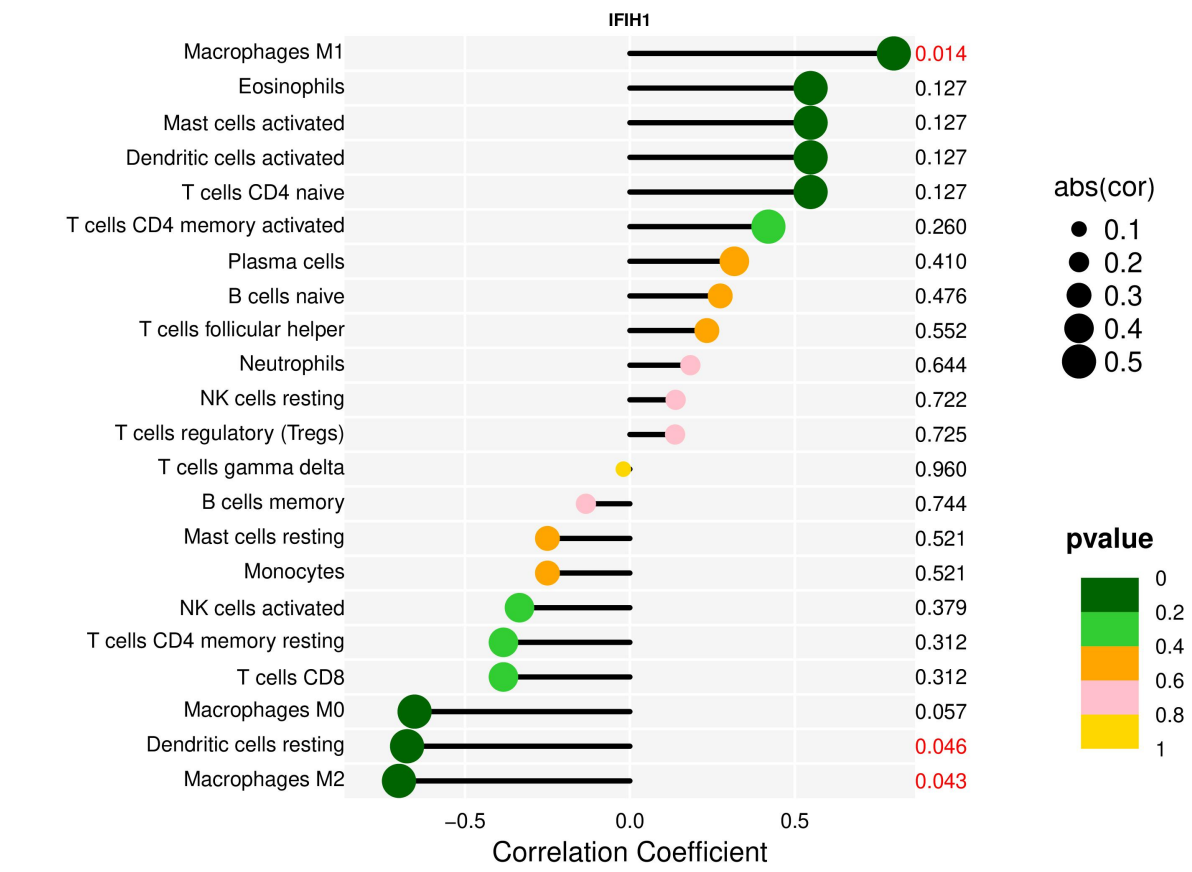

B

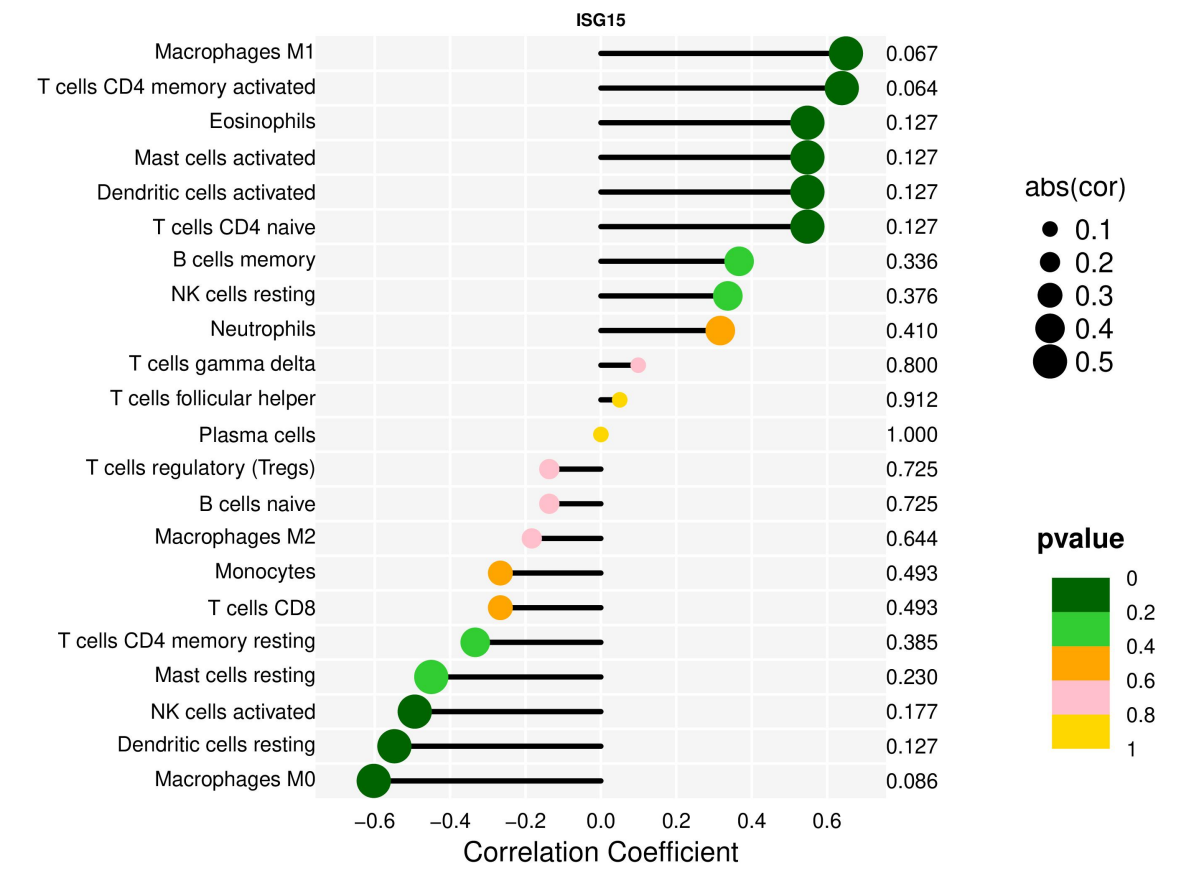

Supplement: Supplementary file 2 — Supplementary Material 2: Supplementary Figure 2. Correlation analysis of IFIH1 and ISG15 with immune cell infiltration. (A) Correlation between IFIH1 expression and immune cell subset abundance. (B) Correlation between ISG15 expression and immune cell subset abundance. [file 13075_2026_3764_MOESM2_ESM.pdf]

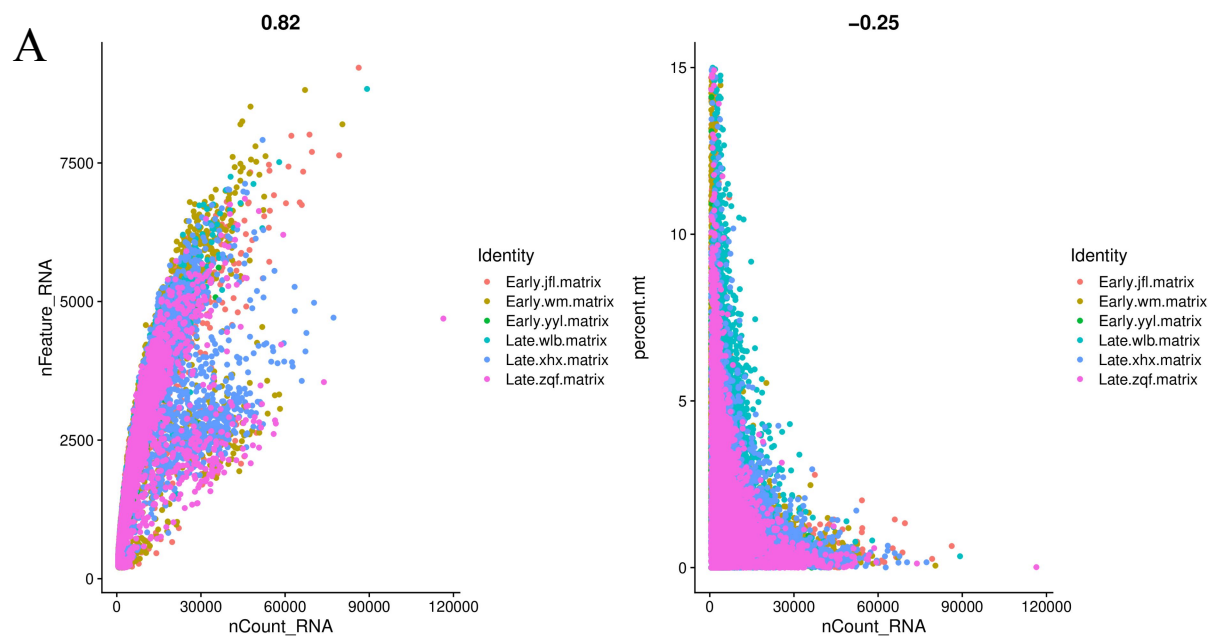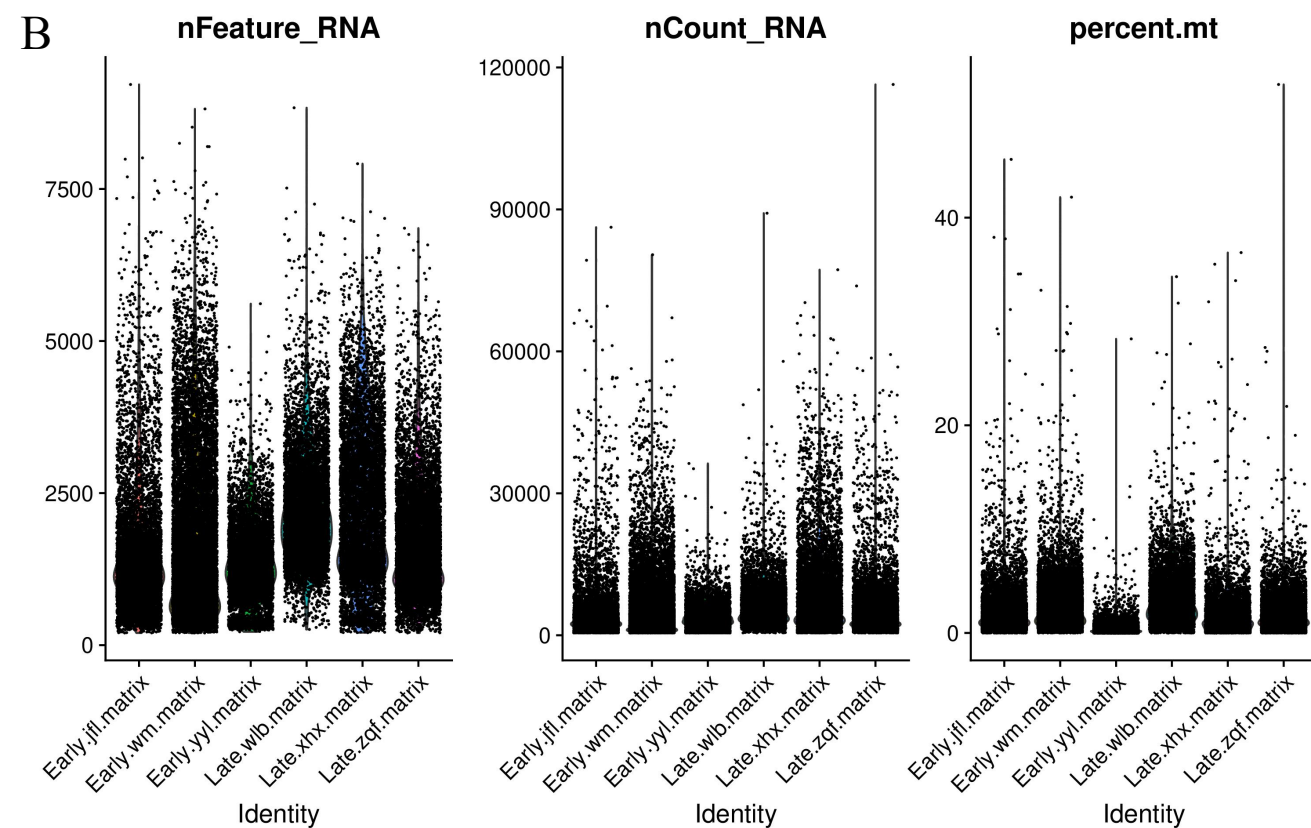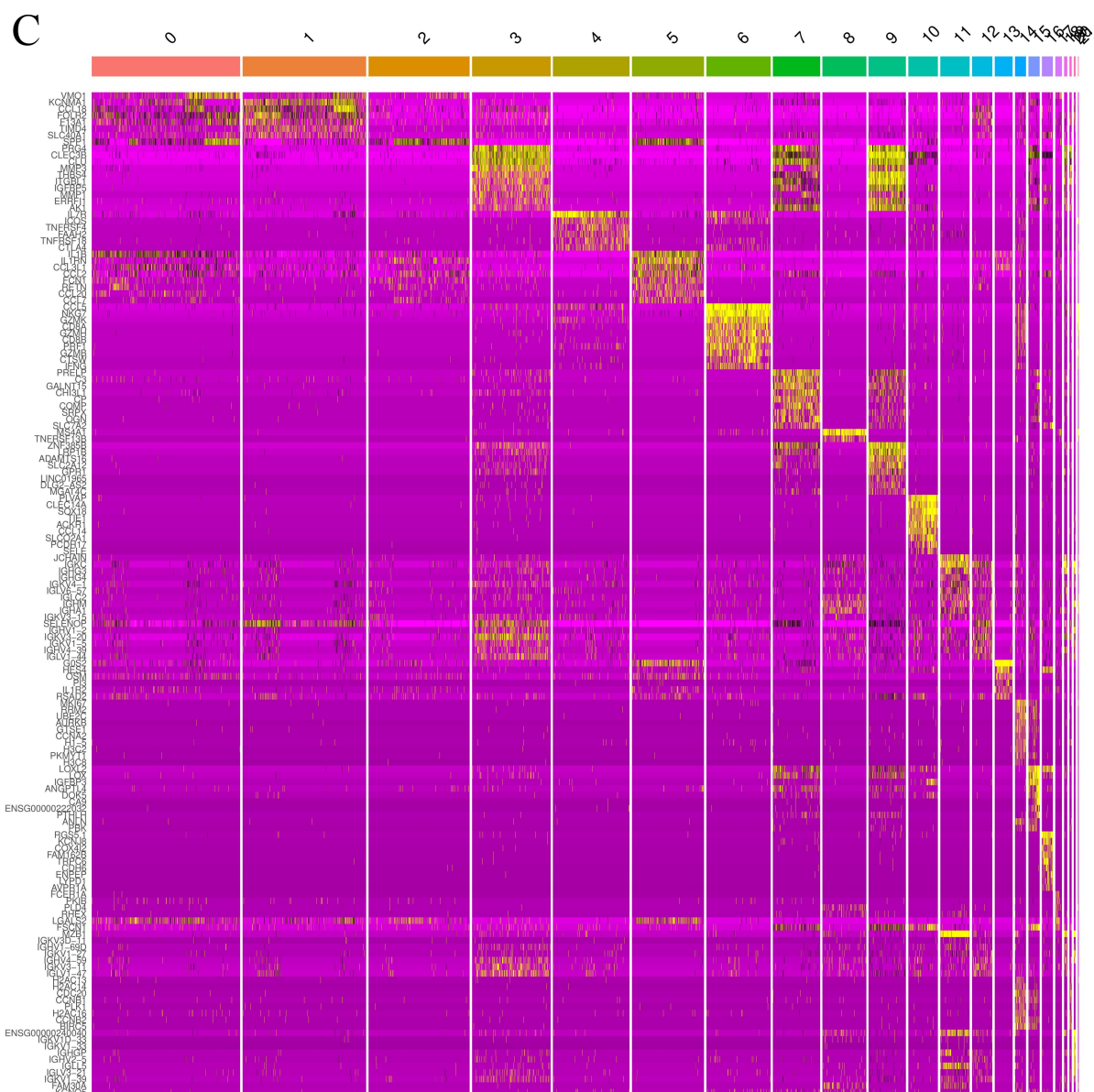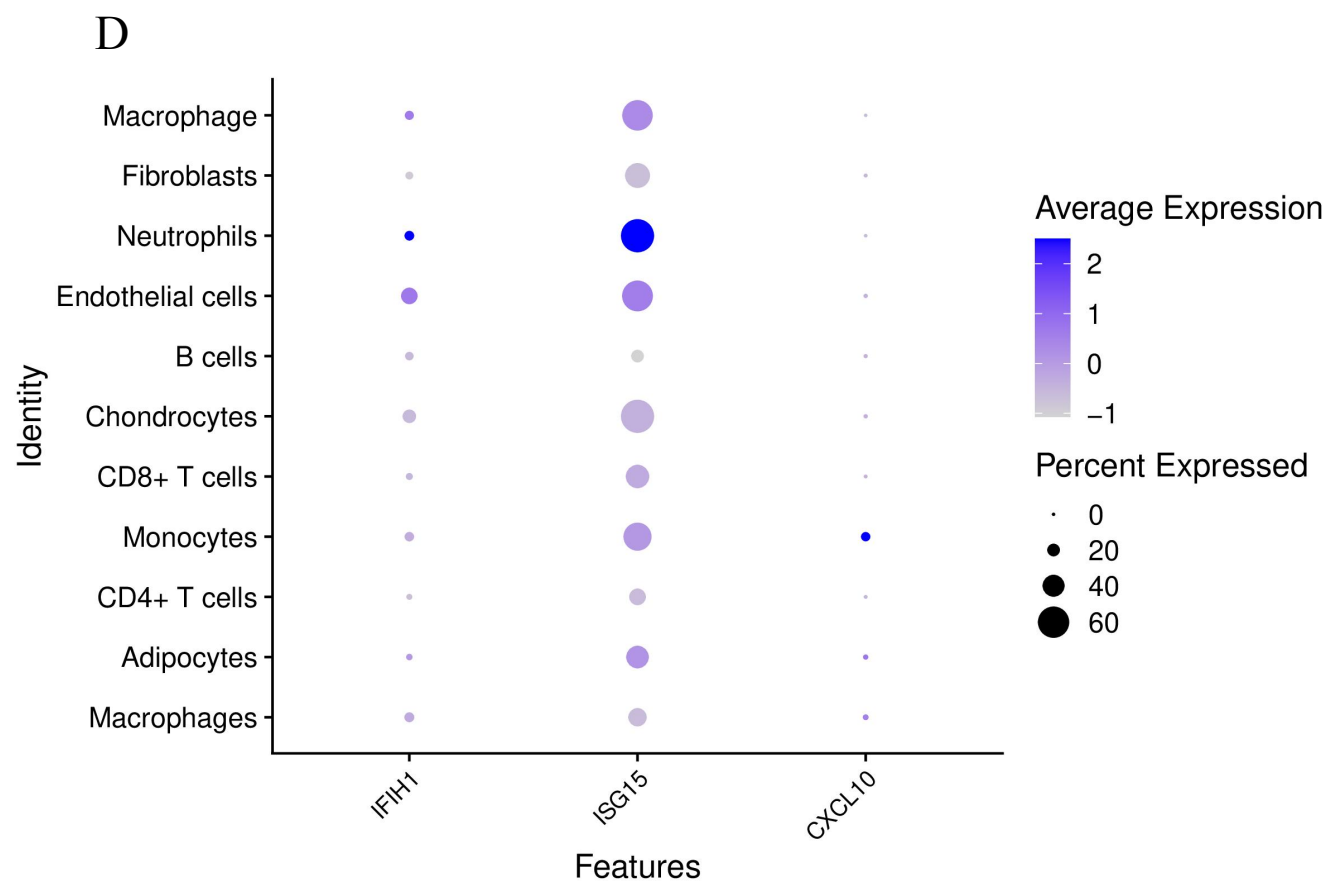

Supplement: Supplementary file 3 — Supplementary Material 3: Supplementary Figure 3. Quality control and cell type identification for scRNA-seq. (A) Quality control scatter plots showing nFeature_RNA vs. nCount_RNA (left) and percent.mt vs. nCount_RNA (right). (B) Quality control violin plots showing the distribution of nFeature_RNA, nCount_RNA, and percent.mt across 6 samples. (C) Heatmap showing expression of classical marker genes used for cell type identification. (D) Dot plot showing expression of IFIH1, ISG15, and CXCL10 across all cell types. [file 13075_2026_3764_MOESM3_ESM.pdf]

A

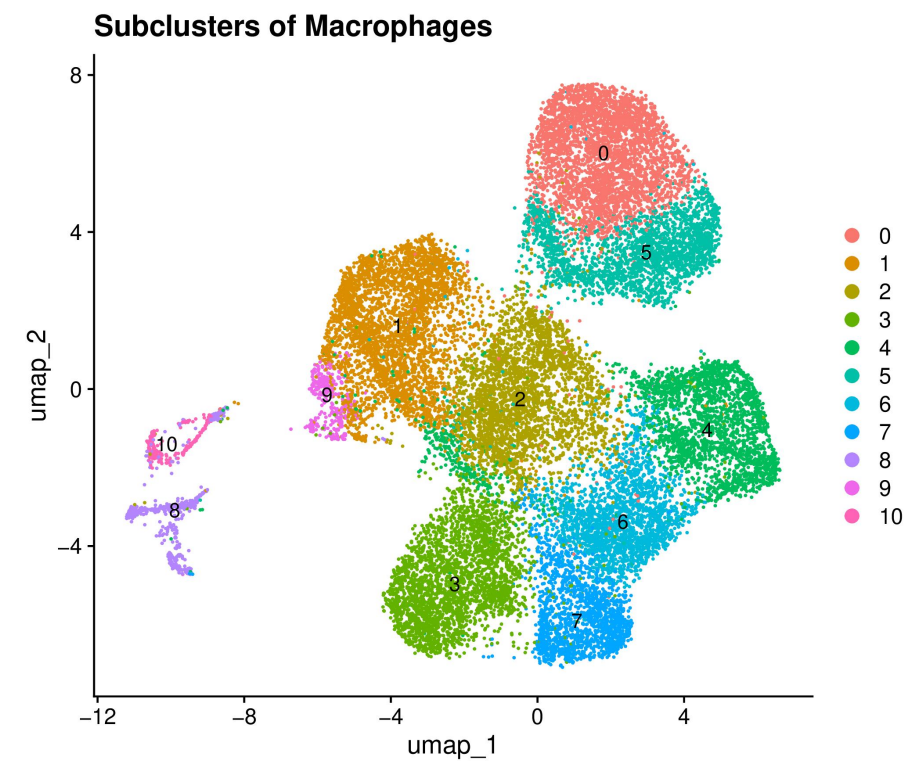

B

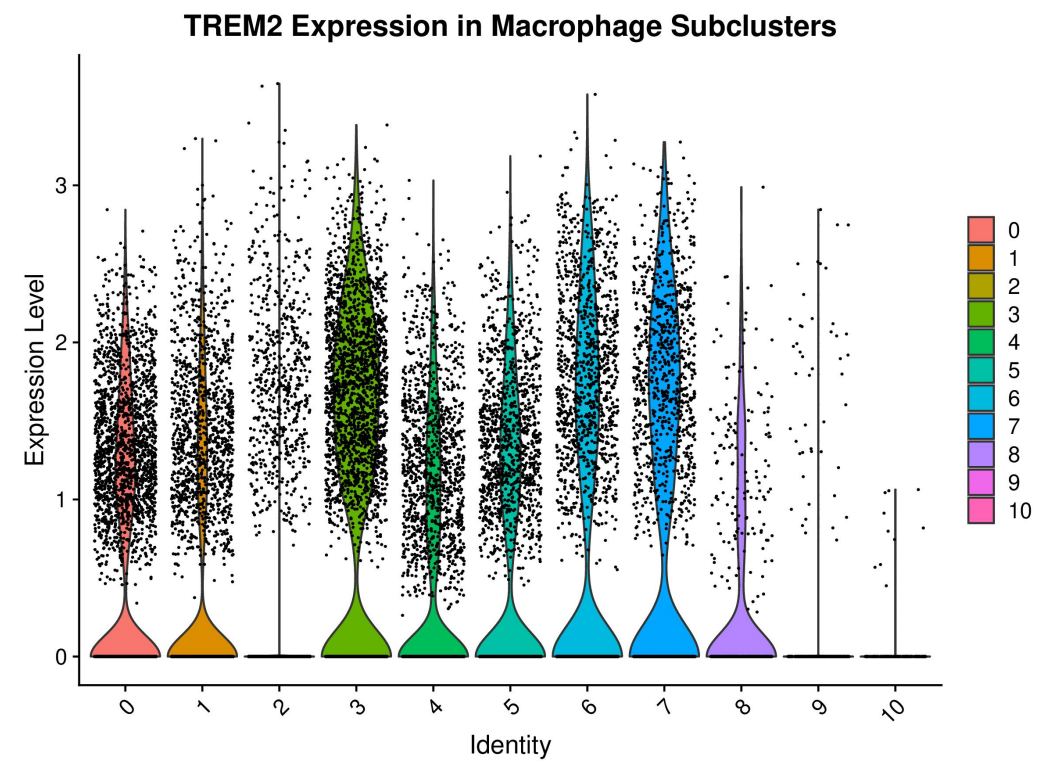

C

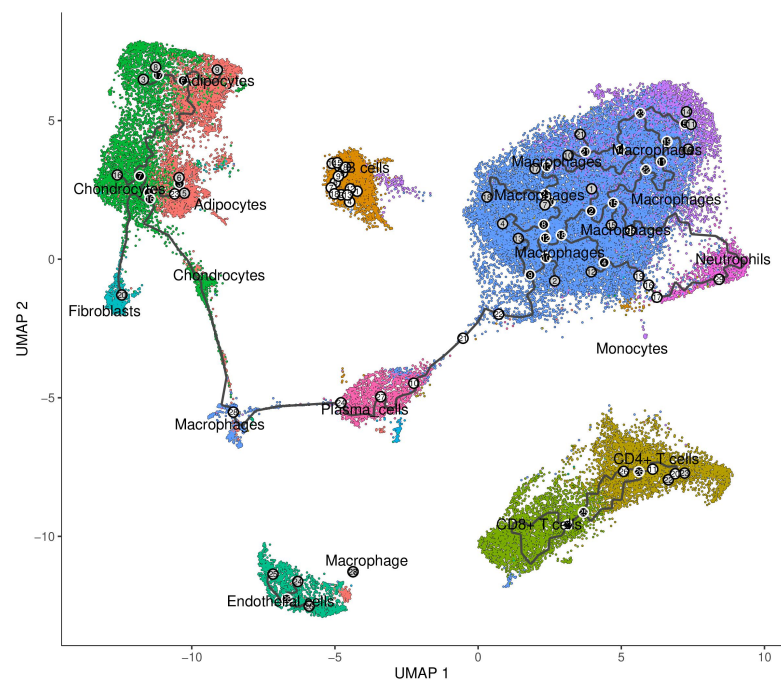

D

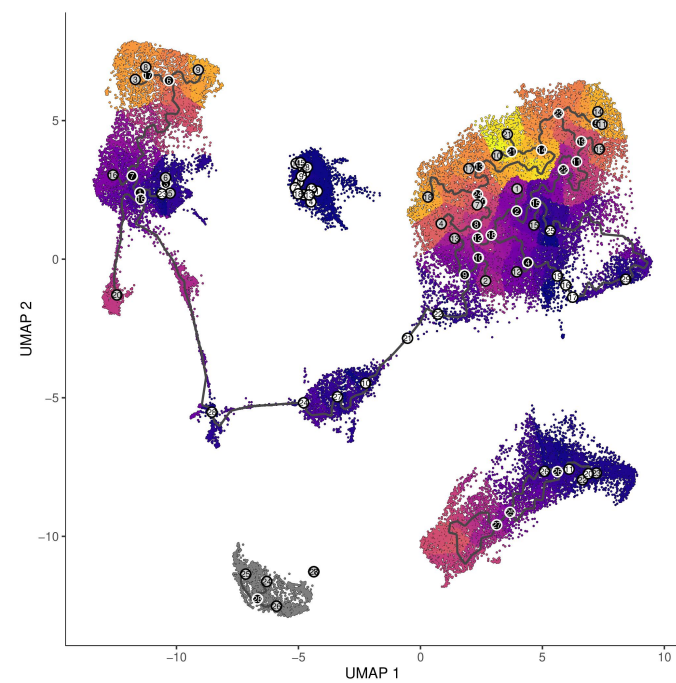

E

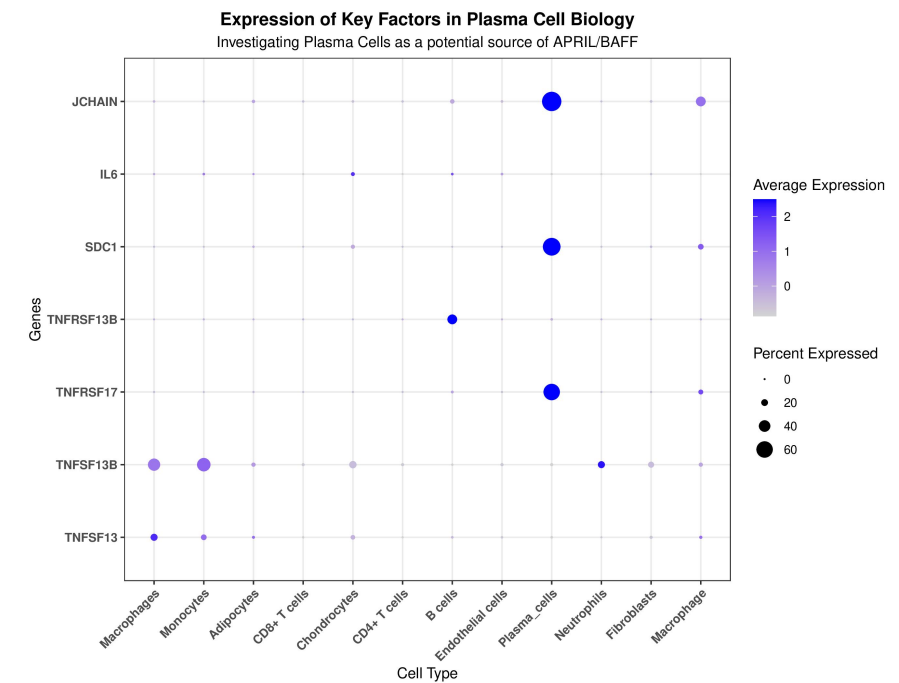

Supplement: Supplementary file 4 — Supplementary Material 4: Supplementary Figure 4. Macrophage subset analysis and B cell/plasma cell trajectory. (A) UMAP visualization of macrophage re-clustering showing 11 subclusters. (B) Violin plot showing TREM2 expression across macrophage subclusters. (C) Monocle3 trajectory analysis showing differentiation relationships of all cell types. (D) Monocle3 trajectory analysis colored by pseudotime values. (E) Dot plot showing expression of key factors in BAFF/APRIL signaling pathway: ligands (TNFSF13/APRIL, TNFSF13B/BAFF) primarily expressed in macrophages/monocytes, and receptors (TNFRSF17/BCMA, TNFRSF13B/TACI) primarily expressed in B cells/plasma cells. [file 13075_2026_3764_MOESM4_ESM.pdf]

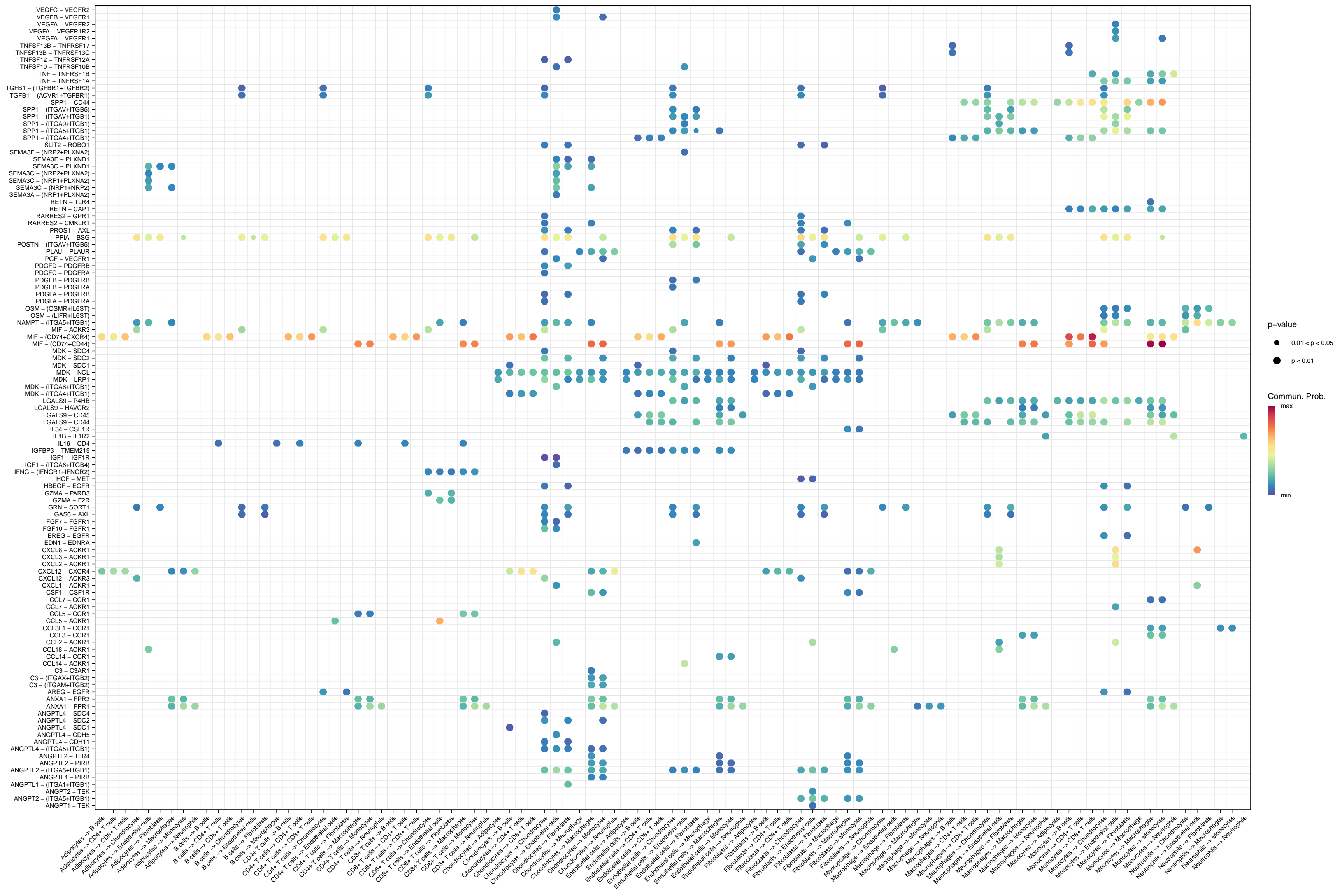

Supplement: Supplementary file 5 — Supplementary Material 5: Supplementary Figure 5. Overview of cell-cell communication network. Dot plot heatmap showing all significant ligand-receptor interactions between cell type pairs. Dot color represents communication probability (red = high, blue = low), and dot size represents statistical significance. [file 13075_2026_3764_MOESM5_ESM.pdf]

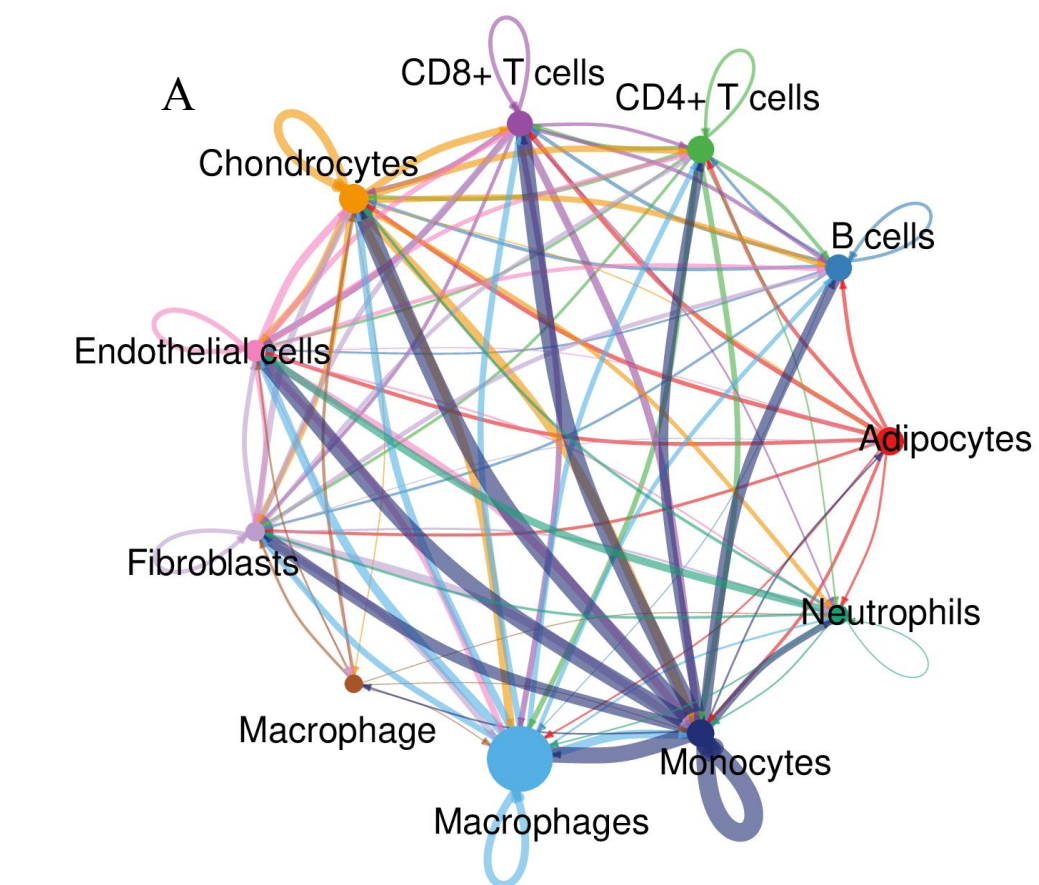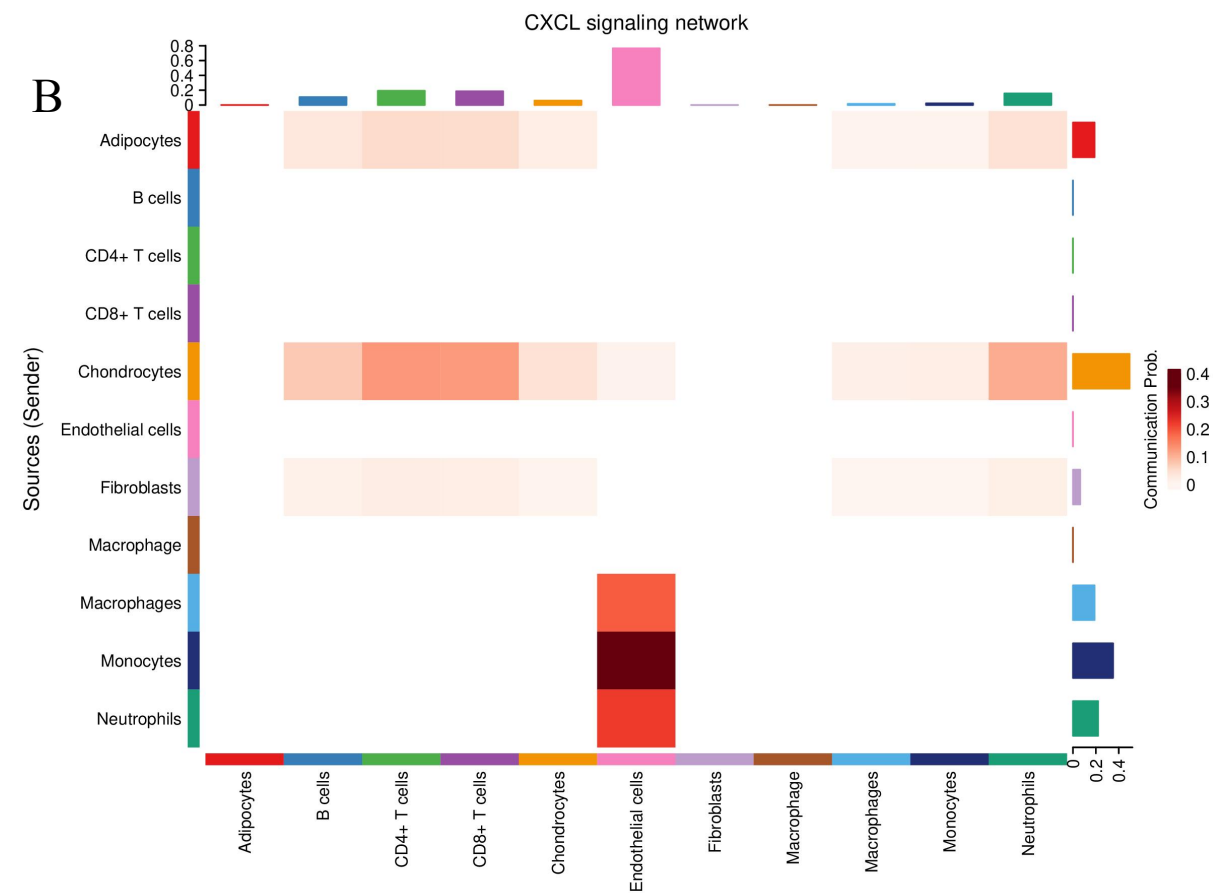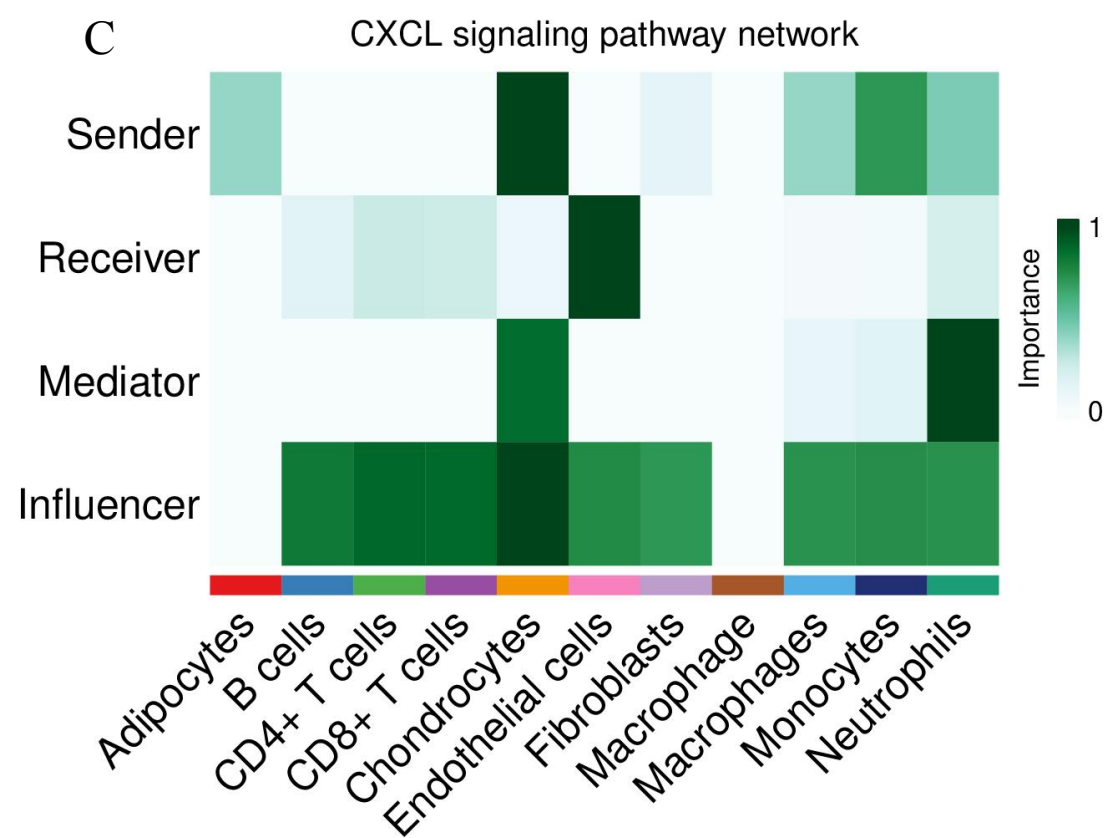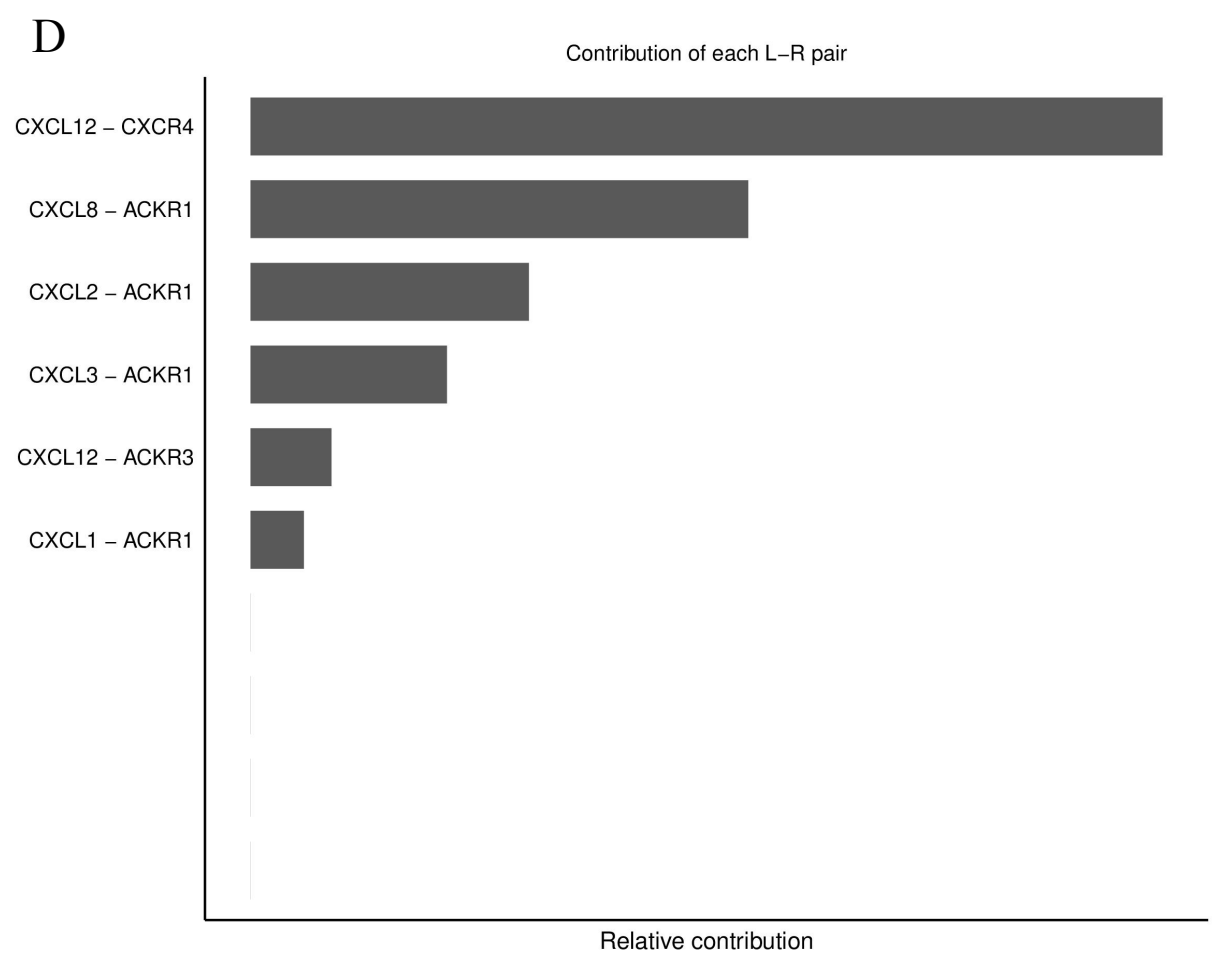

Supplement: Supplementary file 6 — Supplementary Material 6: Supplementary Figure 6. Detailed analysis of CXCL signaling pathway. (A) Circle plot showing cell-cell interactions within the CXCL signaling pathway network. (B) Heatmap showing communication probability between signal senders (rows) and receivers (columns) in the CXCL pathway. (C) Centrality analysis showing the role of each cell type as Sender, Receiver, Mediator, and Influencer in CXCL signaling. (D) Bar plot showing the relative contribution of each ligand-receptor pair to CXCL signaling, with CXCL12-CXCR4 showing the greatest contribution. [file 13075_2026_3764_MOESM6_ESM.pdf]
